# Anti-Obesity Effect of Fermented *Panax notoginseng* Is Mediated *Via* Modulation of Appetite and Gut Microbial Population

**DOI:** 10.3389/fphar.2021.665881

**Published:** 2021-07-26

**Authors:** Na Rae Shin, Shambhunath Bose, Yura Choi, Young-Mi Kim, Young-Won Chin, Eun-Ji Song, Young-Do Nam, Hojun Kim

**Affiliations:** ^1^ Department of Rehabilitation Medicine of Korean Medicine, Dongguk University, Goyang, South Korea; ^2^ College of Pharmacy and Research Institute of Pharmaceutical Sciences, Seoul National University, Seoul, South Korea; ^3^ Research Group of Healthcare, Korea Food Research Institute, Wanju-gun, South Korea

**Keywords:** appetite, gut microbiota, fermentation, obesity, *Panax notoginseng*

## Abstract

*Panax notoginseng* (PN) is a traditional herbal medicine containing several active compounds such as saponins and ginsenosides with many therapeutic applications including anti-obesity activity. Fermentation by lactic acid bacteria has the potential to metabolize ginsenosides to more active forms. This study examined whether fermentation has any benefits on the protective effects of a PN extract against obesity using a high-fat diet (HFD)-fed mouse model. PN was fermented with *Lactobacillus plantarum* which exhibited high β-glucosidase activity. Upon fermentation, the PN extract exhibited an altered ginsenoside profile, a dramatic increase in the lactate level. Treatment of the HFD group with fermented PN (FPN), but not PN, decreased both the food and calorie intake significantly, which was consistent with the more potent suppressing effects of FPN than PN on the signaling pathways involved in appetite and energy intake. The PN treatment also modulated the gut microbial composition. The PN and FPN treatment groups showed clear differences in the population of gut microbiota. The relative abundance of Bacteroidetes, Erysipelotrichaceae, *Coprococus,* and *Dehalobacterium* were significantly higher in the FPN group then the normal, HFD, and XEN groups. Furthermore, the relative abundances of *Akkermansia, Dehalobacterium,* Erysipeliotrichaceae and *parpabacteroides* were significantly higher in the FPN group than the PN group, but the relative abundances of *Allobaculum*, Erysipelotrichi and Erysipelotrichale were significantly lower. The relative abundance of *Bacteroides* and *Lactococcus* was significantly higher and lower, respectively in the PN and FPN groups than the HFD group. In conclusion, the altered ginsenoside and organic acid’s profile, and altered gut microbial composition are believed to be the major factors contributing to the anti-obesity properties of FPN.

## Introduction

Obesity is a common metabolic disease caused by excess caloric intake and lower energy expenditure and is now a serious health challenge to the global population (Ngu et al., 2014). Obesity is closely associated with the onset and development of other complications, diseases, and disorders, including chronic inflammation, type 2 diabetes, insulin resistance, cardiovascular disease, neurodegenerative disorder, cancer, and aging (Hoyt et al., 2014).

Traditional herbal medicines have been used for centuries in Asian countries to treat chronic physiological disorders, such as obesity and diabetes (Yang et al., 2010). *Panax notoginseng* (Burkill) F.H.Chen (PN) is a species of the genus *Panax*, which is also known as “sānqī” in Chinese. PN has a wide range of therapeutic applications because of its many beneficial pharmacological properties, including antioxidant, anti-inflammatory, hypoglycemic, and anti-hyperlipidemic activities (Zhao et al., 2017).

The main active components of PN encompass several types of saponins, including notoginsenoside R1 and ginsenosides Rb1, Rg1, and Rd (Uzayisenga et al., 2014). These ginsenosides have multiple pharmacological efficacies on neuronal, cardiovascular, and immune systems, as well as neuroprotective, anticancer, and antidiabetic activities (Mohanan et al., 2018). The nature of the sugar chains of the ginsenosides is a major factor in determining their functionality. The ginsenosides mentioned above can be transformed by human intestinal bacteria and by exogenous lactic acid bacteria (Bae et al., 2004). The metabolism of ginsenosides by the gut microbiota is influenced mainly by their composition in the host (Kim K.-A. et al., 2013). The bioconversion of ginsenosides to smaller deglycosylated forms may alter their biological activities and physiological actions. For example, a report showed that ginsenosides Rh1 and Rh2, which are transformed from ginsenoside Rg3 by *Bifidobacterium longum*, have anti-obesity effects (Bae et al., 2002; Hwang et al., 2007; Gu et al., 2013). Several studies have focused on the microbial transformation of ginsenosides by the β-glucosidase enzyme of lactic acid bacteria (LAB), such as *Lactobacillus plantarum*, *L. delbrueckii*, *L. fermentum*, *B. longum*, and *Leuconostoc mesenteroides* (Kim et al., 2010; Park et al., 2017). Moreover, PN saponins (ginsenosides Rb1, Rb2, Rd, and notoginsenoside R1) are also metabolized by the glycosidases (β-glucosidase and β-xylosidase) produced by the intestinal microbiota (Niu et al., 2013; Kim, 2018). LAB can produce organic acids such as lactic and acetic acid by metabolizing glucose in the fermentation of carbohydrate substrates including plant-based products (Hamada et al., 1992; Abdel-Rahman et al., 2013). The degree of LAB-mediated fermentation can be determined by quantitative measurement of organic acids which are important parameters for monitoring the bacterial growth and activity (Izco et al., 2002). Although some studies have evaluated the effects of fermented ginseng root on obesity (Uzayisenga et al., 2014; Li et al., 2018), only a few studies have addressed the therapeutic potential of fermented *Panax notoginseng* (FPN) (Lin et al., 2010).

The gut microbiota, which plays a vital role in regulating the metabolism and maintaining the homeostasis of the nutrient and energy levels of the host, is closely linked to obesity (Turnbaugh et al., 2006). Previous studies have suggested that an imbalance in the gut microbial composition influences the development of obesity by interfering with several factors, including energy harvest from the diet, systemic inflammation, and fat deposition (Bajzer and Seeley, 2006; Tsai and Coyle, 2009). Although the administration of ginseng modulates the gut microbial composition in obese women (Song et al., 2014), to the best of the authors’ knowledge, no study has assessed the impact of fermentation on the anti-obesity effects of PN. In the present study, using a high-fat diet (HFD)-fed mice model, this study examined the anti-obesity properties of PN fermented with *L. plantarum*, which showed high β-glucosidase enzymatic activity. This study also investigated whether the beneficial impacts of FPN are mediated via modulation of the gut microbial population.

## Materials and Methods

### Selection of Candidate Lactic Acid Bacterial Starter Strains

The candidate lactic acid bacterial-starter strains isolated from fermented herbs, food, and rice wine were screened for their β-glucosidase enzymatic activity. The bacteria were grown in Lactobacilli MRS broth (Difco Laboratories, Detroit, MI, United States) at 37°C for 24 h. The bacterial suspensions were then centrifuged at 12,000 *g* for 3 min, and the supernatants were discarded. The bacterial pellets were washed three times with PBS (pH 7.4), and the cells were re-suspended in PBS. A portion of this suspension was then mixed with 3 mM 4-nitrophenyl β-D-glucopyranoside (p-NPG, Sigma Aldrich, St. Louis, MO, United States) at a 1:1 (v/v) ratio, and the mixture was incubated at 37°C for 10 min. The β-glucosidase enzymatic activity of the samples was measured colorimetrically at 420 nm using a microplate reader (Spectramax Plus, Molecular Devices, Sunnyvale, CA, United States).

### Preparation of *Panax notoginseng* and Fermented *Panax notoginseng*

The dried root of *Panax notoginseng* (Burkill) F.H.Chen (PN) in powder form was procured from the medical supply store of Dongguk University International Hospital (Ilsan, Goyang-si, Republic of Korea). The PN extracted with 30% ethanol (v/v) and evaporated to dryness using a rotary evaporator (EYELA N-1200A, EYELA, Tokyo, Japan), followed by freeze-drying using a lyophilizer (Bondiro, IlshinBioBase, Dongducheon, Republic of Korea) (For more details, see the supporting data). Finally, the prepared PN with a yield of 22.27% (w/w) was stored at −80°C until further use.

To prepare the fermented *Panax notoginseng* (FPN), three selected LAB strains were incubated in the MRS broth media at 37°C for 24 h. The bacterial suspension was centrifuged at 12,000 *g* for 3 min, after which the supernatant was discarded. The pelleted bacterial cells were washed three times with PBS (pH 7.4), and finally placed in 50 g of lyophilized PN extract reconstituted with 500 ml of distilled water. The bacterial cells were incubated with PN at 37°C for 24 h. The number of viable cells was counted before and after fermentation. The FPN preparation was lyophilized and stored at −80°C for future use.

### Ginsenoside Metabolism Analysis

The ginsenoside biotransformation activity of *L. plantarum* M41 was measured with ginsenoside Rg3. The M41 strain was incubated in MRS broth medium containing 0.2 mg/ml of ginsenoside Rg3 for 24 h at 37°C. The metabolites were measured using the High-performance liquid chromatography (HPLC) system.

### High-Performance Liquid Chromatography-Based Analysis of Lactic Acid, Acetic Acid, and Ginsenoside Rg3

The HPLC system (1260 infinity, Agilent Technologies, Santa Clara, CA, United States) was equipped with an auto-sampler and a UV detector**.** For lactic and acetic acid analysis, the mobile phase was composed of 0.008N sulfuric acid. Separation was achieved on an Aminex-87H column (150 mm, 4.6 mm, Bio-Rad, United States) at 35°C. The flow rate of the mobile phase was maintained at 0.6 ml/min, and the run time was set to 30 min. Detection was performed at 210 nm. Lactic and acetic acid (Sigma Aldrich, St. Louis, MO, United States) were used as standards for the analysis.

For the analysis of ginsenoside Rg3, the mobile phase was composed of (A) water and (B) acetonitrile. Separation was achieved on an Eclipse XDB-C18 column (5 μm, 250 × 4.6 mm. Agilent Technologies, Santa Clara, CA, United States) at 40°C. The flow rate of the mobile phase was maintained at 1 ml/min, and the run time was set to 30 min. Detection was performed at 203 nm. The programmed gradient elution was performed as follows: 50% B (0–12 min), 50–75% B (12–20 min), 75–10% B (20–25 min) and 10% B (25–30 min). For the analysis, ginsenoside Rg3 (Sigma Aldrich, St. Louis, MO, United States) were used as standards.

### Ultra Performance Liquid Chromatography Time of Flight/Mass Spectrometry Analysis

The chromatographic separation of PN or FPN was performed on a Waters Acquity™ UPLC system (Waters Corp., Milford, MA, United States) equipped with a binary solvent delivery system, an auto-sampler, a column heater, and a DAD detector. Separation was achieved on an ACQUITY UPLC®BEH C18 column (2.1 × 100 mm, 1.7 µm; Waters). Lyophilized PN or FPN powders were dissolved in 50% methanol at a final concentration of 10 mg/ml and filtered through a 0.22-mm-membrane syringe filter (Sartorius Amtsgericht, Gottingen, Germany). Standard Rg1, Rg2, Rb1, Rd, and Rg3S ginsenoside (Chengdu Biopurify Phytochemicals Ltd, Sichuan, China) solutions were prepared individually with methanol at a final concentration of 100 μg/ml. Two microlitres aliquots of filtered PN or FPN were then injected into the UPLC system. The mobile phase was composed of (A) 0.1% formic acid in water (v/v) and (B) 0.1% formic acid in acetonitrile (v/v), whicj was delivered at a flow rate of 0.45 ml/min using the following programmed gradient elution: 8% B (0–0.5 min), 8–10% B (0.5–8 min), 10–11% B (8–10 min), 11–12% B (10–20 min), 12–14% B (20–30 min), 14–21% B (30–40 min), 21–23% B (40–45 min), 23–45% B (45–50 min), 45–75% B (50–53 min), 75–8% B (53–53.5 min), and 8% B (53.5–55 min). The column temperature was maintained at 40°C. Detection was achieved at 203 nm.

MS analysis was performed on a Waters XEVO G2 Q-TOF mass spectrometer (Waters) equipped with an electrospray ionization (ESI) source operating in negative ionization mode. The collision energy was set at 40 eV. The capillary and cone voltages were 2800 and 40 V, respectively. The gas flow rates for the cone and desolvation gas (nitrogen) were set at 30 and 800 L/h, respectively. The source and desolvation temperatures were 120 and 800°C, respectively. The mass spectral data were recorded in the range of m/z 100–2,000.

### Animal Studies

Four-week-old female C57BL/6 mice (17.7 ± 0.8 g) were obtained from DBL Inc. (Eumseong-gun, Republic of Korea). The animals were provided with a high-fat diet (HFD, 60% kcal from fat, Research Diets Inc., New Brunswick, NJ, United States) supplemented with 20% fructose in drinking water for two weeks to induce obesity. The normal group of mice (NOR) were fed AIN 93G diet (16% Kcal from fat, Research Diets Inc.). The HFD-induced mice were then divided randomly into four groups (two to three animals per cage) and treated orally with water as the vehicle according to the following specifications: treated with Xenical (XEN) (*n* = 8, 10 mg/kg/day), PN (*n* = 7, 400 mg/kg/day), FPN (*n* = 7, 400 mg/kg/day), and HFD alone (*n* = 7, treated with water). The animals in the NOR group (*n* = 8) were also treated orally with water. The treatments were carried out five times per week for nine weeks (Supplementary Figure 1). The body weights of the animals were measured once a week and the food intake of the animals were recorded three times a week. Fresh stool samples were collected one week before sacrifice of and stored at −80°C for future microbial analyses. The animal study was approved by the Institutional Animal Care and Use Committee of Dongguk University (IACUC-2018-005).

After completing of the treatment schedule, the animals were sacrificed with a combination of Zoletil (Tiletamine-zolazepam, Virbac, Carros, France)-Rompun (xylazine-hydrochloride, Bayer, Leverkusen, Germany) anesthesia (1:1, v/v). Blood samples were then collected from the central aorta and transferred into a BD Vacutainer (BD, Franklin Lakes, NJ, United States). The visceral and perigonadal adipose tissues were excised quickly, washed in ice-cold PBS (pH 7.4), dried, weighed and stored at −80°C until used. A portion of the liver, fat, hypothalamus, and intestinal tissues were also excised rapidly, washed and submerged in RNAlater solution (Invitrogen, Taastrup, Denmark). The samples were then stored at −80°C until for RNA isolation. The total fat weight was estimated as the combined weight of the visceral and perigonadal adipose tissues. The blood samples were allowed to clot for 2 h at room temperature and then were centrifuged at 3,000 *g* for 15 min. The sera were separated and stored at −80°C for further analysis.

### Measurement of the Serum and Hepatic Triglycerides Content

The serum TG level was determined using a commercial enzymatic assay kit (Asan Pharmaceutical Co., Seoul, Republic of Korea) according to the manufacturer’s instructions.

The stored liver tissues (100 mg) were homogenized in 1 ml of distilled water using a tissue homogenizer (OMNI International, Warrenton, VA, United States). The homogenate was extracted with 5 ml of a chloroform-methanol (2:1 v/v) mixture and centrifuged at 7,000 *g* for 5 min. The chloroform layer was aspirated, dried, and resolved by isopropanol. The hepatic TG content was determined using a kit, as mentioned above for serum TG analysis.

### Tissue RNA Extraction and Real-Time Quantitative Polymerase Chain Reaction

The total RNA was extracted from the tissues using a TRIzol reagent (Invitrogen Life Technologies, Carlsbad, CA, United States) according to the manufacturer’s instructions. For RT-qPCR, an equal quantity of each RNA sample (1 μg) was reverse transcribed to synthesize the cDNA using the oligo deoxythymine 18-mer ([dT]_18_) primer (Thermo Scientific, Waltham, Massachusetts, United States) and cDNA RT PreMix kit (Bioneer, Daejeon, Republic of Korea). The qPCR analysis was performed on a Light Cycler 480™ platform (Roche Applied Science, Mannheim, Germany) in a 96-well plate using an SYBR Green Real-time PCR Master Mix (Toyobo, Osaka, Japan). Subsequently, melting curve analysis was performed to verify the purity and specificity of the amplicon. The data processing and analyses were performed using dedicated Light Cycler software supplied by the instrument manufacturer (Roche Applied Science, version 1.2). The C_t_ values were normalized using glyceraldehyde-3-phosphatase dehydrogenase (GAPDH) as a housekeeping gene, and the relative gene expression was quantified using the standard 2^−ΔΔCt^ method**.** The Supplementary Table 2 lists the primer sequences.

### Fecal Microbial DNA Extraction and Sequencing

Microbial genomic DNA from fecal samples was isolated using a QIAamp stool DNA mini kit (QIAGEN, Hilden, Germany) according to the kit manufacturer’s instructions. The PCR of the V1-V2 region of the 16S rRNA genes was performed using a thermal cycler system (Bio-Rad, Hercules, CA, United States), after which the amplicons were purified using a LaboPass PCR purification kit (COSMO GENTECH, Seoul, Republic of Korea). An equimolar concentration of each amplicon from the different samples was pooled to equal proportions based on their molecular weights and purified using Agencourt AMPure XP PCR purification beads (Agencourt Bioscience, Beverly, MA, United States). The DNA concentration and quality were checked using a BioAnalyzer 2100 microfluidic device (Agilent Technologies, Inc., Santa Clara, CA, United States) using a DNA 100 lab chip (Agilent Technologies, Inc.). The mixed amplicons were then amplified on sequencing beads by emulsion PCR (BioRad). The sequencing reactions were performed using an ion torrent personal genome machine (Ion PGM, Thermo Scientific) according to the manufacturer’s instructions.

### Analysis of Sequenced Data

The sequences were then assigned to the operational taxonomic units [OTUs; 97% identity; Greengenes database (http://greengenes.lbl.gov/cgi-bin/nph-index.cgi)], followed by the selection of representative sequences using the Quantitative Insights into Microbial Ecology (QIIME) software package (Caporaso et al., 2010). The taxa with differing abundances among the groups were identified by LDA Effect Size (LEfSe) analysis using an online program (http://huttenhower.sph.harvard.edu/galaxy) (Segata et al., 2011). Permutational multivariate analysis of variance (PERMANOVA) was conducted to compare the relative distances between the groups. The raw data of the sequence is available in the European Nucleotide Archive with the accession numbers ERP119284.

### Statistical Analysis

The data are expressed as the means ± SEM unless stated otherwise. The statistical significance was evaluated using a student’s t-test, one-way analysis of variance (ANOVA) followed by a Tukey’s post-hoc test, or two-way ANOVA followed by a Sidak’s multiple comparisons test. The strength of the relationships between the parameters was assessed using the two-tailed Pearson’s correlation test. The correlations were considered significant only when the absolute value of the Pearson’s correlation coefficient (r) was greater than 0.5.

## Results

### Selection of the Lactic Acid Bacteria Strain as a Starter for *Panax notoginseng* Fermentation

**FIGURE 1:**
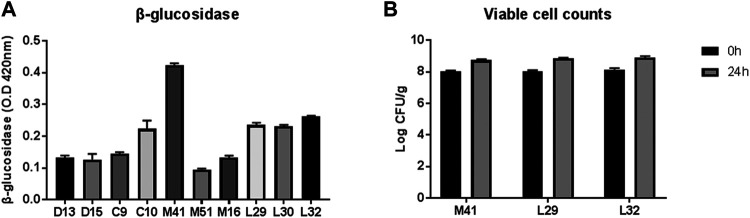
Selection of lactic acid bacteria for PN fermentation. **(A)** Profile of β-glucosidase activity of the candidate-isolated fermented food. **(B)** Viable cell counts before and after PN fermentation with three candidates.

Three out of ten LAB strains that showed relatively higher β-glucosidase activity (M41, L29, and L32) were screened for their efficiency in PN fermentation (Figure 1A). Upon incubation with PN for 24 h, the viable counts of all three LAB candidates were increased significantly, as revealed by a two-way ANOVA test, but the number was similar in the three groups (Figure 1B). Finally, the M41 strain that exhibited the highest β-glucosidase activity and was identified as *L. plantarum* by 16s rRNA gene sequencing was chosen as the candidate to ferment PN.

### Chemical Characterization of *Panax notoginseng* and Fermented *Panax notoginseng*

**FIGURE 2:**
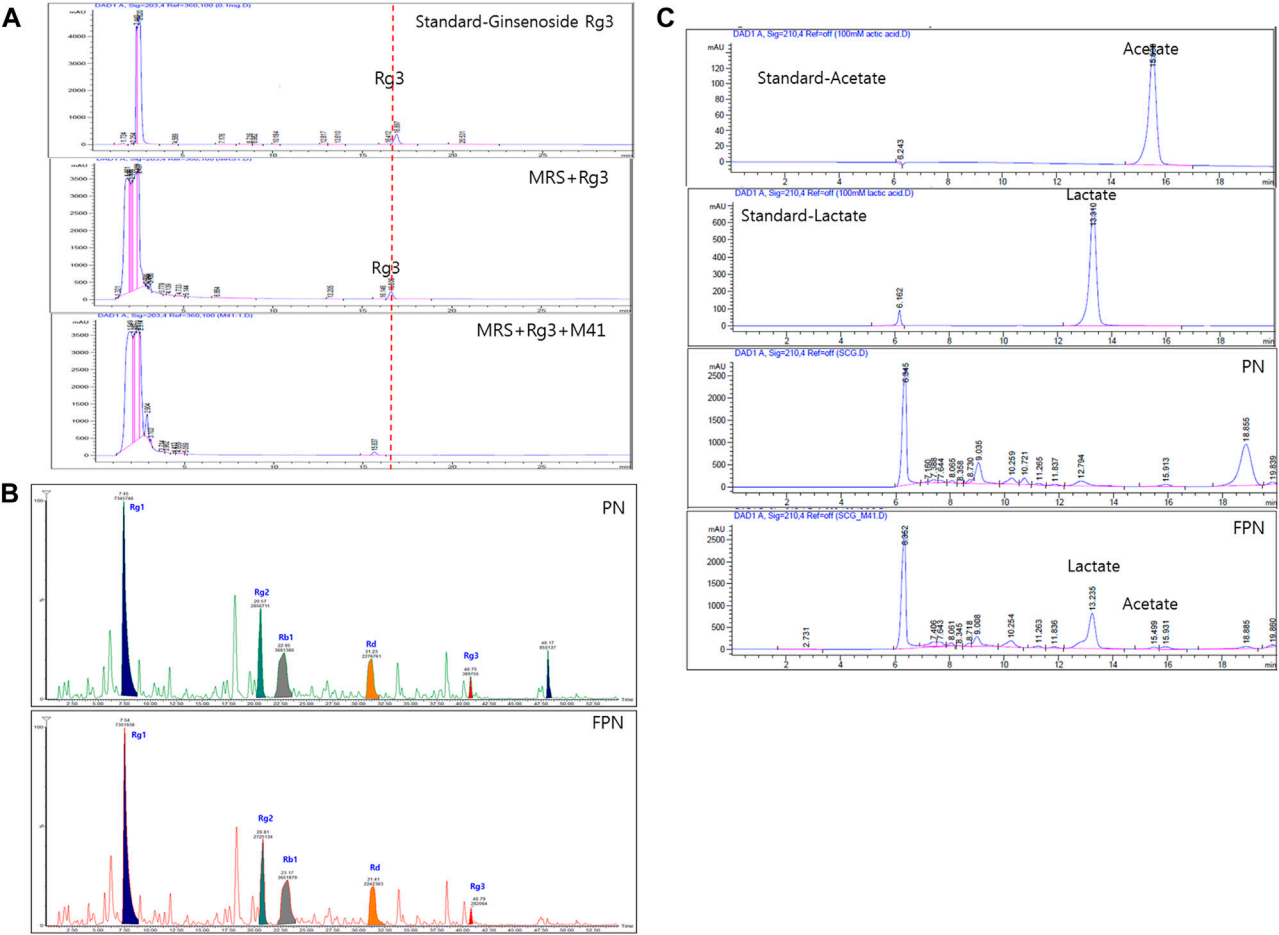
Profile analysis of active compounds before and after fermentation of PN. **(A)** Ginsenoside Rg3 was metabolized by M41 which was measured by HPLC. **(B)** UPLC-TOF-MS of ginsenosides and **(C)** HPLC with UV detector analysis of lactic and acetic acid.

The ginsenoside conversion capacity of the M41 strain was measured, by HPLC. When the M41 was incubated in MRS broth for 24 h, it metabolized ginsenoside Rg3 (Figure 2A). The profile of ginsenosides analyzed with UPLC-TOF MS showed that both PN and FPN contained ginsenosides Rg1, Rg2, Rb1, Rd and Rg3 (Figure 2B; Supplementary Figure 2). Interestingly, the peak area of Rg3S decreased after PN fermentation (28% decline, Figure 2B). HPLC fingerprinting analysis showed that the concentrations of acetic acid and lactic acid of PN increased from undetectable levels to 28.0 and 246.3 mM, respectively after fermentation (Figure 2C; Supplementary Table 2).

### Effects of *Panax notoginseng* and Fermented *Panax notoginseng* on the Body Weight, Fat Weights, and Food and Calorie Intake in High Fat Diet-Fed Mice

**FIGURE 3:**
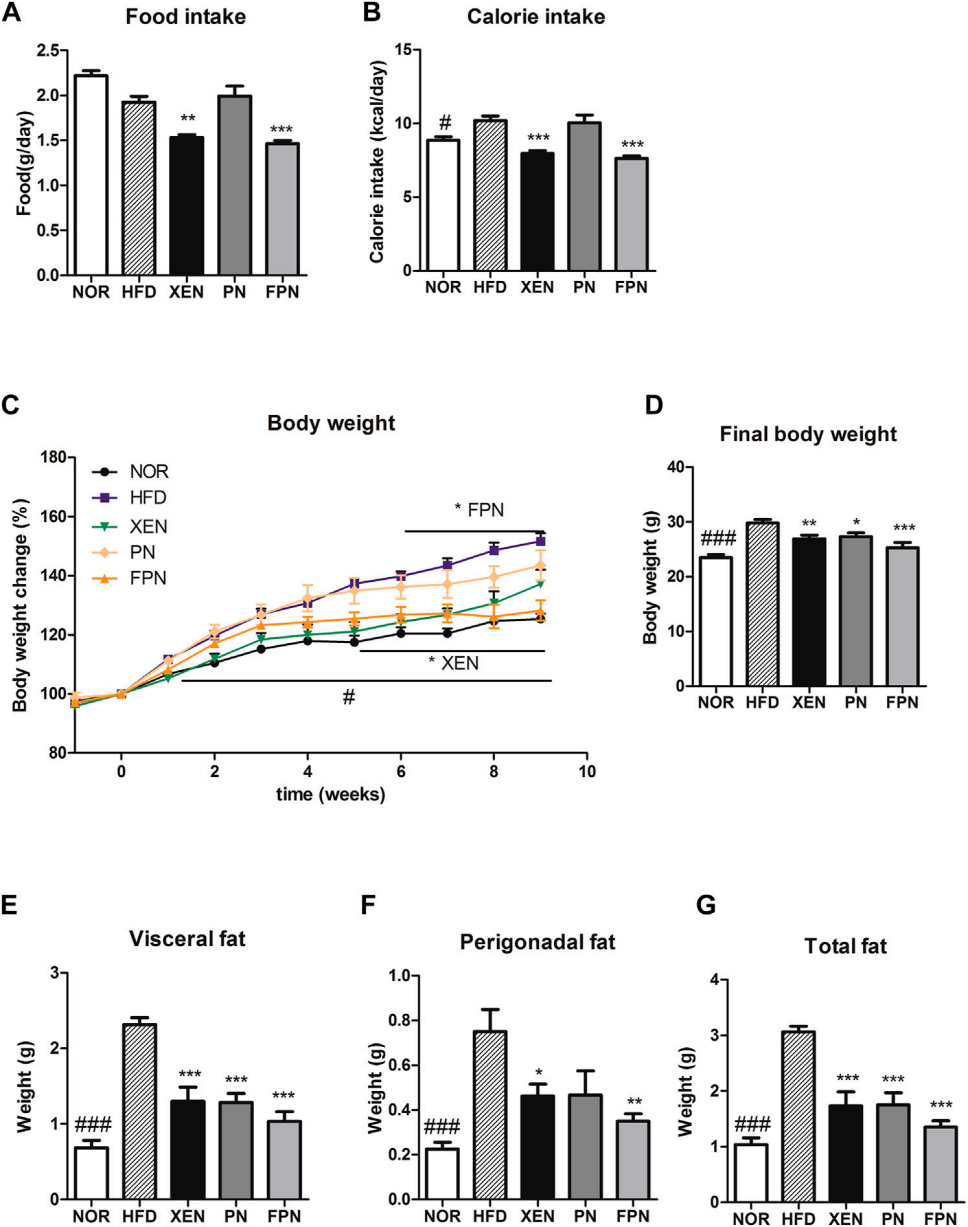
Effect of PN and FPN on the body and fat weight and food intake in a HFD-induced mouse model after treatment for 9 weeks. **(A)** Food intake, **(B)** calorie intake, **(C)** body weight, **(D)** final body weight, **(E)** visceral fat weight, **(F)** perigonadal fat weight, and **(G)** total fat weight. The data are expressed as the mean ± SEM (*n* = 7–8); #*p* < 0.05; ###*p* < 0.001; NOR *vs.* HFD group. *p < 0.05; **p < 0.01; ***p < 0.001; HFD group *vs.* treatment groups (XEN, PN and FPN).

At the end of the experimental schedule on week seven, the food intake remained unchanged, but the calorie intake was significantly higher in the HFD than in the NOR groups (*p* < 0.05, Figures 3A,B). On the other hand, the food and calorie intakes in the HFD group decreased significantly after a treatment with XEN and FPN, but not PN. The body weight and weights of the visceral, perigonadal, and total fat were significantly higher in the HFD group than the NOR group (*p* < 0.001, Figures 3C–G). The body weight of the HFD-fed animals was reduced by all three treatments, but more significantly by FPN (*p* < 0.001) than XEN (*p* < 0.01) or PN (*p* < 0.05, Figure 3D). All the treatments also caused a significant decrease in the weights of the visceral and total fats (*p* < 0.001) in the HFD group (Figures 3E,G). The weight of perigonadal fat in the HFD-fed animals was reduced significantly by both XEN (*p* < 0.05) and FPN (*p* < 0.01), but not PN (Figure 3F).

### *Panax notoginseng* and Fermented *Panax notoginseng* Modulated Lipid Metabolism in the High Fat Diet Group

**FIGURE 4:**
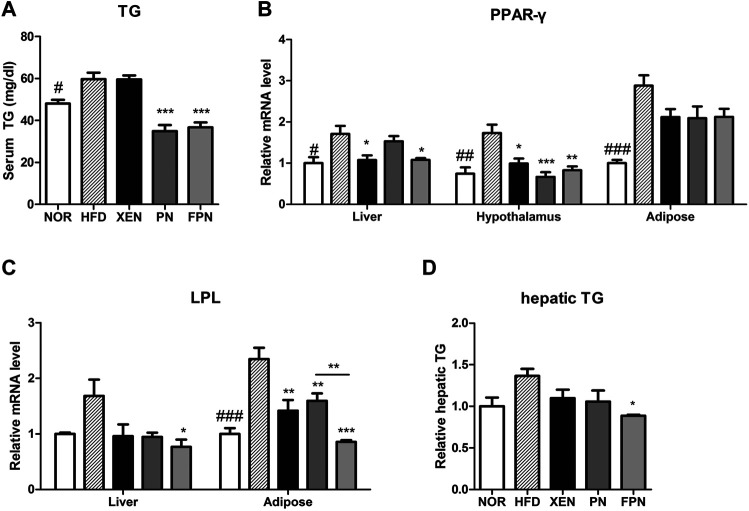
Effect of PN and FPN on the lipid metabolism of HFD-induced mouse model after treatment for 9 weeks. The TG was measured in **(A)** serum and **(D)** liver. The expression of the genes of interest in the liver, hypothalamus, and adipose tissue was determined by RT-PCR. The relative gene expressions of **(B)** PPAR-γ and **(C)** LPL which play an important role in lipid metabolism were measured. The data are expressed as the mean ± SEM (*n* = 7–8); #*p* < 0.05; ###*p* < 0.001; NOR *vs.* HFD group. *p < 0.05; **p < 0.01; ***p < 0.001; HFD group *vs.* treatment groups (XEN, PN and FPN).

The serum TG concentration, mRNA levels of PPAR-γ in the liver, hypothalamus, and adipose tissue, and the mRNA level of LPL in the adipose tissue were significantly higher in the HFD group than in the NOR group (Figure 4). The serum TG level in the HFD-fed animals was reduced significantly by both PN and FPN treatments but not by XEN (Figure 4A). The levels of the PPAR-γ and LPL gene expression in the hypothalamus and adipose tissue, respectively, decreased significantly in the HFD group after all three treatments (Figures 4B,C). The hepatic expression of the PPAR-γ gene was downregulated significantly in the HDF group by XEN and FPN, but not by PN. On the other hand, TG levels and the expression of the LPL gene in the liver were reduced significantly in the HFD-fed animals only by FPN (Figures 4C,D, *p* < 0.05).

### *Panax notoginseng* and Fermented *Panax notoginseng* Influenced Appetite

**FIGURE 5:**
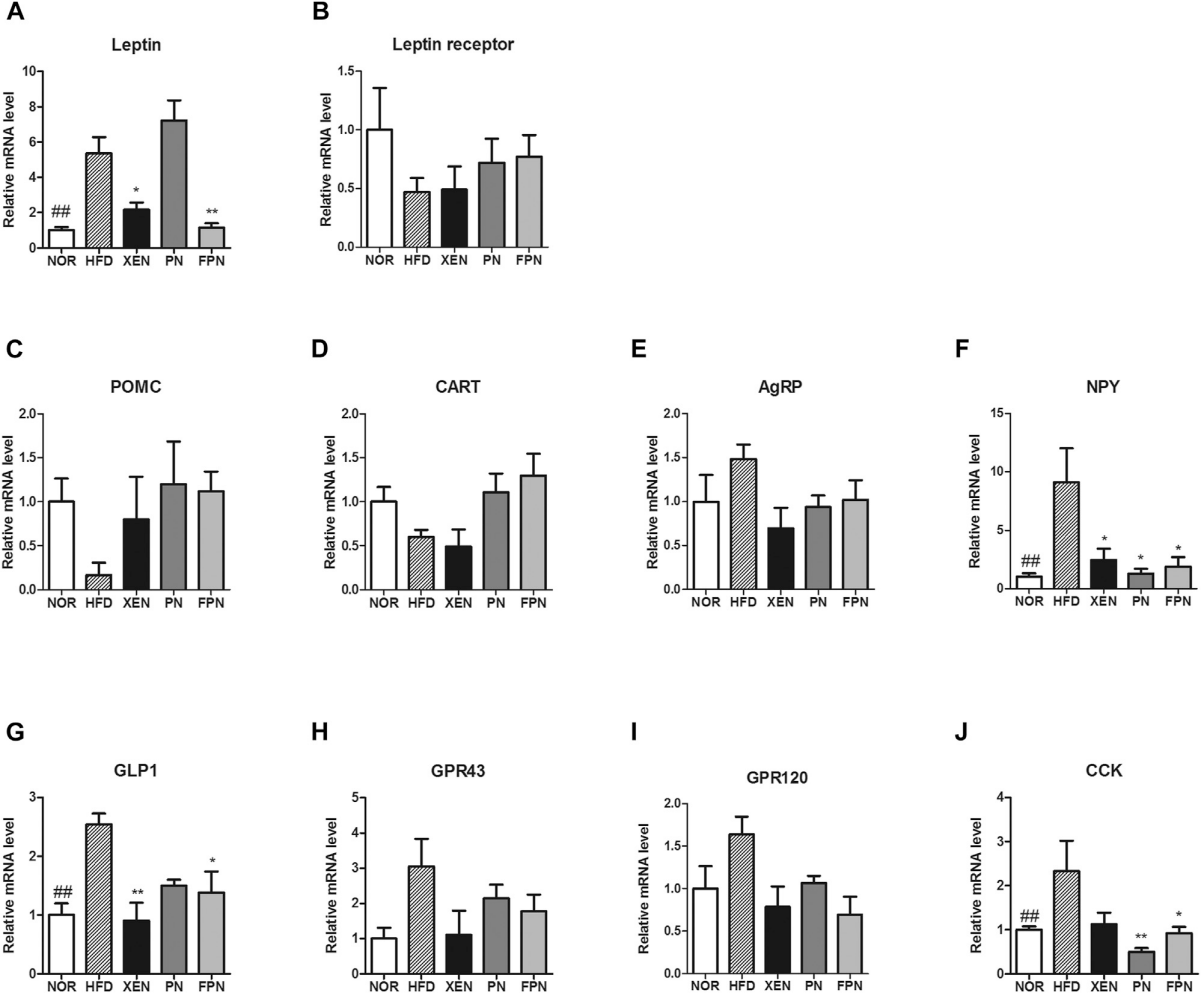
Effect of PN and FPN on gene expressions of the satiety hormone leptin, appetite regulators, and gut hormones in HFD induced mouse model after treatment for 9 weeks. The expressions levels of the gene of interest in the hypothalamus, adipose tissue, and intestine were determined by RT-PCR. The relative gene expression of **(A)** leptin in adipose tissue was measured. The relative gene expressions of appetite regulators **(B)** leptin receptor **(C)** POMC, **(D)** CART, **(E)** AgRP, and **(F)** NPY in the hypothalamus was determined. The relative gene expression of gut hormones **(G)** GLP1, **(H)** CPR43, **(I)** GPR 120, and **(J)** CCK in the colon was measured. The data are expressed as the mean ± SEM (*n* = 7–8); ##*p* < 0.005; NOR *vs.* HFD group. *p < 0.05; **p < 0.01; HFD group *vs.* treatment groups (XEN, PN and FPN).

The expression levels of the leptin gene in the adipose tissue, NPY gene in the hypothalamus, and the GLP-1 and CCK genes in the gut were significantly higher in the HFD group than the NOR group (Figures 5A,F,G,J). The gene expression levels of the leptin receptor, POMC, CART, and AgRP in the hypothalamus were similar among the four groups (Figures 5B–E). The mRNA levels of leptin and GLP1 were reduced significantly in the HFD-fed animals by the XEN and FPN treatments, but not by PN (Figures 5A,G). While, the expression of the NPY gene in the hypothalamus was suppressed significantly by the XEN, PN and FPN treatments (Figure 5F). On the other hand, the colonic mRNA level of CCK in the HFD group was reduced significantly by the PN and FPN treatments, but not by XEN (Figure 5J). The levels of GPR43 and GPR120 gene expression in the colon were also similar among the four groups (Figures 5H,I).

### *Panax notoginseng* and Fermented *Panax notoginseng* Exerted Anti-inflammatory Effects

**FIGURE 6:**
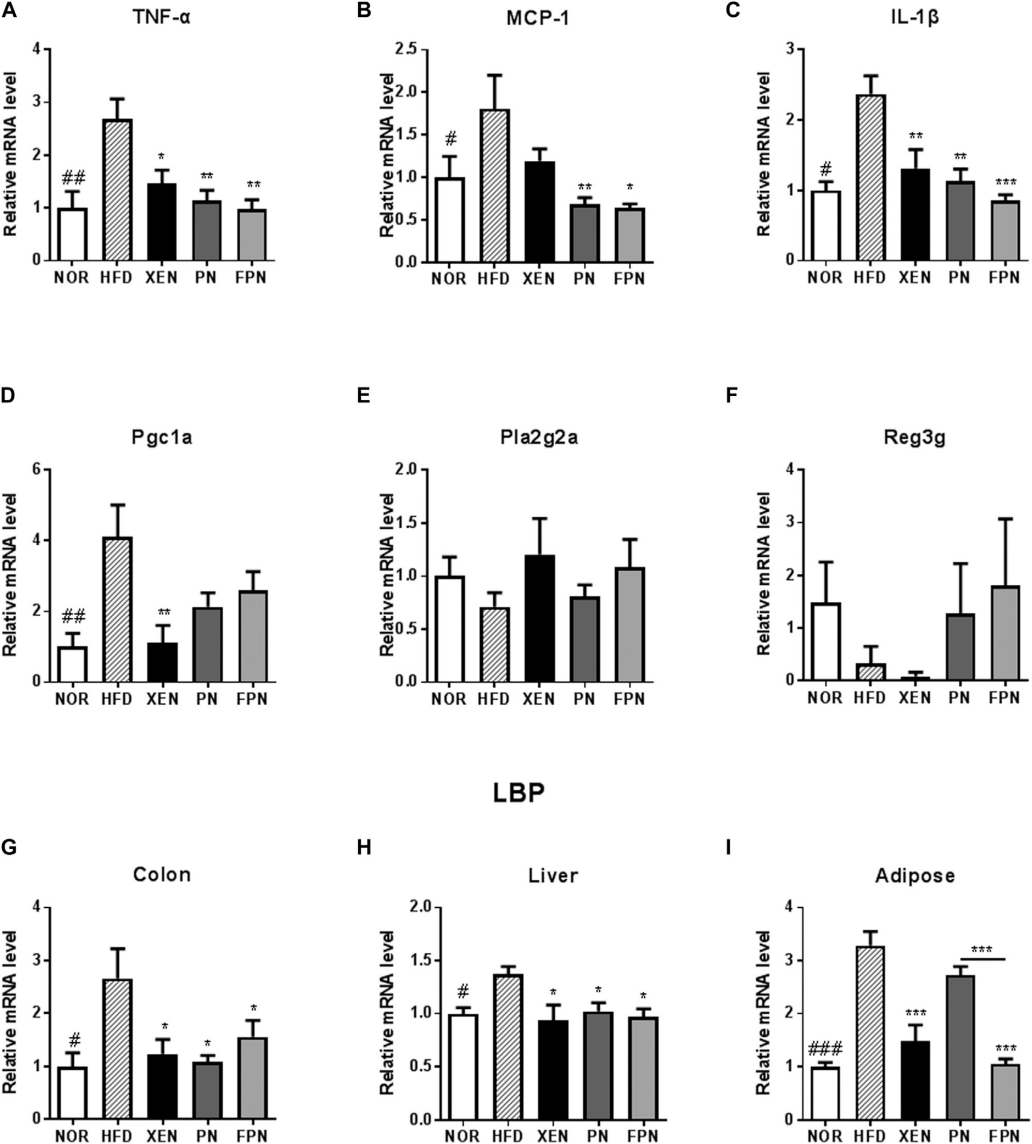
Effect of PN and FPN on the gene expression of inflammatory mediators and antimicrobial peptides in a HFD-induced mouse model after treatment for 9 weeks. Expressions of the genes of interest in the intestine, liver, and adipose were determined by RT-PCR. The relative gene expression of the inflammatory mediators **(A)** TNF-α, **(B)** MCP-1, and **(C)** IL-1β as well as relative gene expressions of anti-microbial peptides **(D)** Pgc1α, **(E)** Pla2g2α, and **(F)** Reg3γ were measured in the colon tissue. The relative gene expressions of LPS binding protein (LBP) was measured in **(G)** colon, **(H)** liver, and **(I)** adipose tissue. The data are expressed as the mean ± SEM (*n* = 7–8); #*p* < 0.05; ##*p* < 0.005; ###*p* < 0.001; NOR *vs.* HFD group. *p < 0.05; **p < 0.01; ***p < 0.001; HFD group *vs.* treatment groups (XEN, PN and FPN).

The colonic mRNA levels of TNF-α, MCP-1, IL-1β, PGC1a, and LBP, as well as the levels of LBP mRNA in the liver and adipose tissue, were significantly higher in the HFD group than in the NOR group (Figure 6). On the other hand, the histology evaluation of the colon using H & E stainning neither revealed any change in the tissue architecture nor showed any prominent signs of inflammation in response to the HFD treatment (Supplementary Figure 3). The colonic expressions of TNF-α, IL-1β, and LBP and the hepatic expression of the LBP genes in the HFD-fed animals were downregulated significantly by the three scheduled treatments (Figures 6A–C,G–I). The mRNA level of MCP1 in the colon of the HFD group was reduced significantly by PN and FPN but not by XEN (Figure 6B). In contrast, the expression of the colonic PGC1a gene was attenuated significantly by XEN but not by PN and FPN (Figure 6D). In contrast, the colonic gene expression levels of Pla2g2a and Reg3g were similar in the four groups (Figures 6E,F). The adipose tissue mRNA level of LBP was reduced significantly by XEN and FPN but not by PN (Figures 6G–I).

### *Panax notoginseng* and Fermented *Panax notoginseng* Influenced Gut Microbial Diversity

**FIGURE 7:**
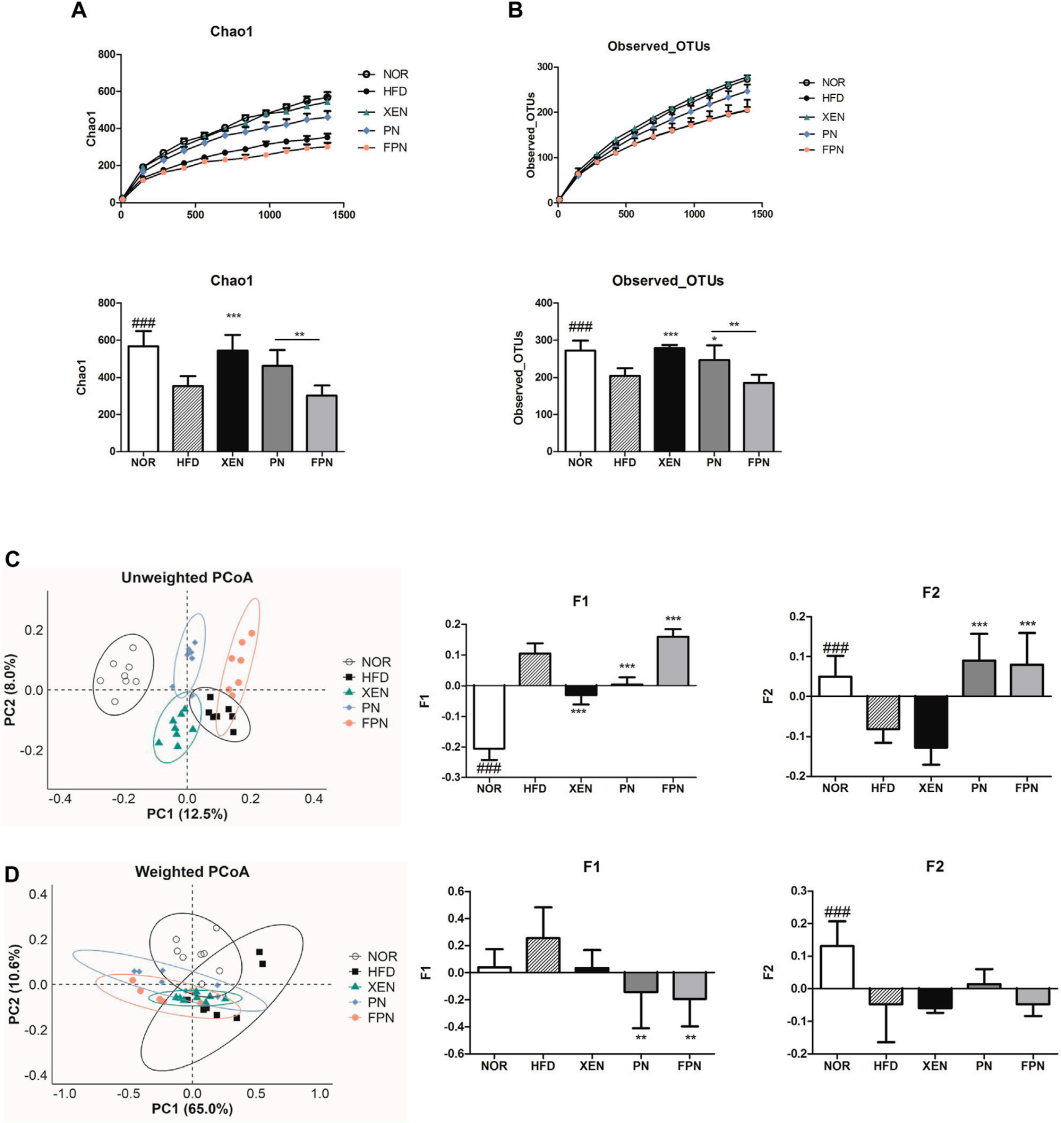
Changes in the diversity and structure of the gut microbiota in response to exposure to PN or FPN in the HFD-induced mouse model. The alpha diversity was measured from **(A)** Chao 1 and **(B)** observed OTUs. PCoA score plot calculated from OTU level by QIIME was subjected to **(C)** unweighted and **(D)** weighted UniFrac analysis. The data are expressed as the mean ± SEM (*n* = 7–8); ###*p* < 0.001; NOR *vs.* HFD group. *p < 0.05; **p < 0.01; ***p < 0.001; HFD group *vs.* treatment groups (XEN, PN and FPN).

Both Chao1 and observed OTUs, which are indices of α-diversity, and measure of the species richness, were significantly lower (*p* < 0.001) in the HFD group than the NOR group (Figures 7A,B). The treatment of HFD-fed animals with XEN, but not PN and FPN, enhanced the Chao1 significantly. Exposure of the HFD group to XEN and PN, but not FPN, increased the observed OTUs significantly. Specifically, a significant difference in Chao 1 and the observed OTUs was observed between the PN and FPN groups. PCoA of the unweighted and weighted methods was used to evaluate the β-diversity, indicating the similarity of the samples. In the unweighted PCoA, each of the aforementioned experimental groups showed a distinct clustering pattern of the gut microbial population (Figure 7C).

The principal component F1 and F2 scores of this analysis were significantly higher and lower, respectively, in the HFD group than the normal group. Treatment of the HFD-fed animals with PN and FPN decreased and increased F1, respectively. Exposure of the HFD group to both PN and FPN increased F2 significantly. In contrast, the weighted PCoA revealed a dispersed distributional pattern of the gut microbial communities among all the experimental groups with noticeable inter-group overlap (Figure 7D). An insignificant increase in F1 and a significant decrease in F2 were observed in the animals in response to HFD-feeding. Treatment of the HFD group with both PN and FPN resulted in a significant decrease in F1. PERMANOVA was conducted to compare the distances between the groups. The HFD group differed significantly from all the other experimental groups except the FPN in unweighted UniFrac (Supplementary Table 3). Furthermore, the distance between the PN and FPN groups also showed significant difference. On the other hand, only the PN group showed significant differences from the NOR and HFD groups in weighted UniFrac analysis.

### Gut Microbial Composition Differed Between *Panax notoginseng* and Fermented *Panax notoginseng* Groups

**FIGURE 8:**
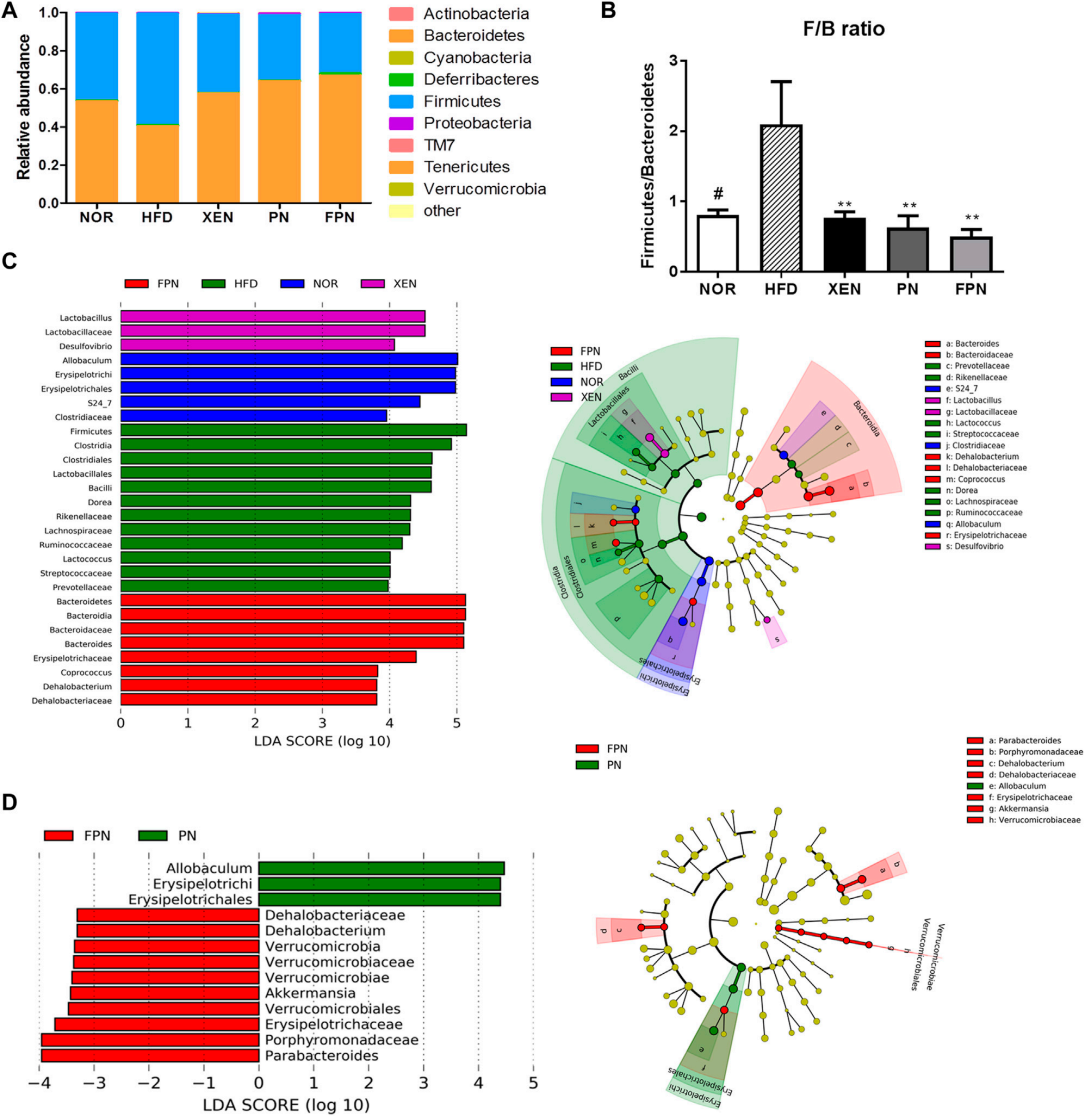
Inter-group variations in the relative abundances of the gut microbial communities in the HFD-induced mouse model after treatment for 9 weeks. **(A)** relative abundances at the phylum level, **(B)** relative abundance of Bacteroidetes and Firmicutes in each group, **(C)** a taxonomic comparison of the bacterial communities among all groups and between **(D)** PN and FPN groups. Significant differences in the LDA scores (*p <* 0.05) were produced among the classes (Kruskal-Wallis test). The threshold of the logarithmic LDA score was >2.0.

At the phylum level, the HFD group showed the highest abundance of Firmicutes (F) and the lowest population of Bacteroidetes (Figure 8A), reflecting the significantly higher Firmicutes/Bacteroidetes (F/B) ratio vs. the NOR group (Figure 8B). Treatment of the HFD group with both PN and FPN reduced the F/B ratio significantly. LEfSe analysis showed that the relative abundance of microbial taxa differed among the groups (Figure 8C). The cladogram from the LEfSe results showed that compared to the HFD group, 11, eight, six, and 11 taxa were higher in the NOR, XEN, PN, and FPN groups, respectively, while 13, five, eight, and 11 taxa were lower. Significant changes in the relative abundances of microbial taxa were observed between the PN and FPN groups (Figure 8D). Allobacuium, Erysipeltrichi, and Etysipelotrichales were higher in the PN group than the FPN group, while Dehalobacteriaceae, Dehalobacterium, Verrucomicrobia, Verrucomicrobiaceae, Verrucomicrobiae, *Akkermansia*, Verrucomicrobiales, Erusipelotrichaceae, Porphyromonadaceae, and *Parabacteriodes* were lower.

### Correlation Between the Gut Microbiota and Appetite Markers

**FIGURE 9:**
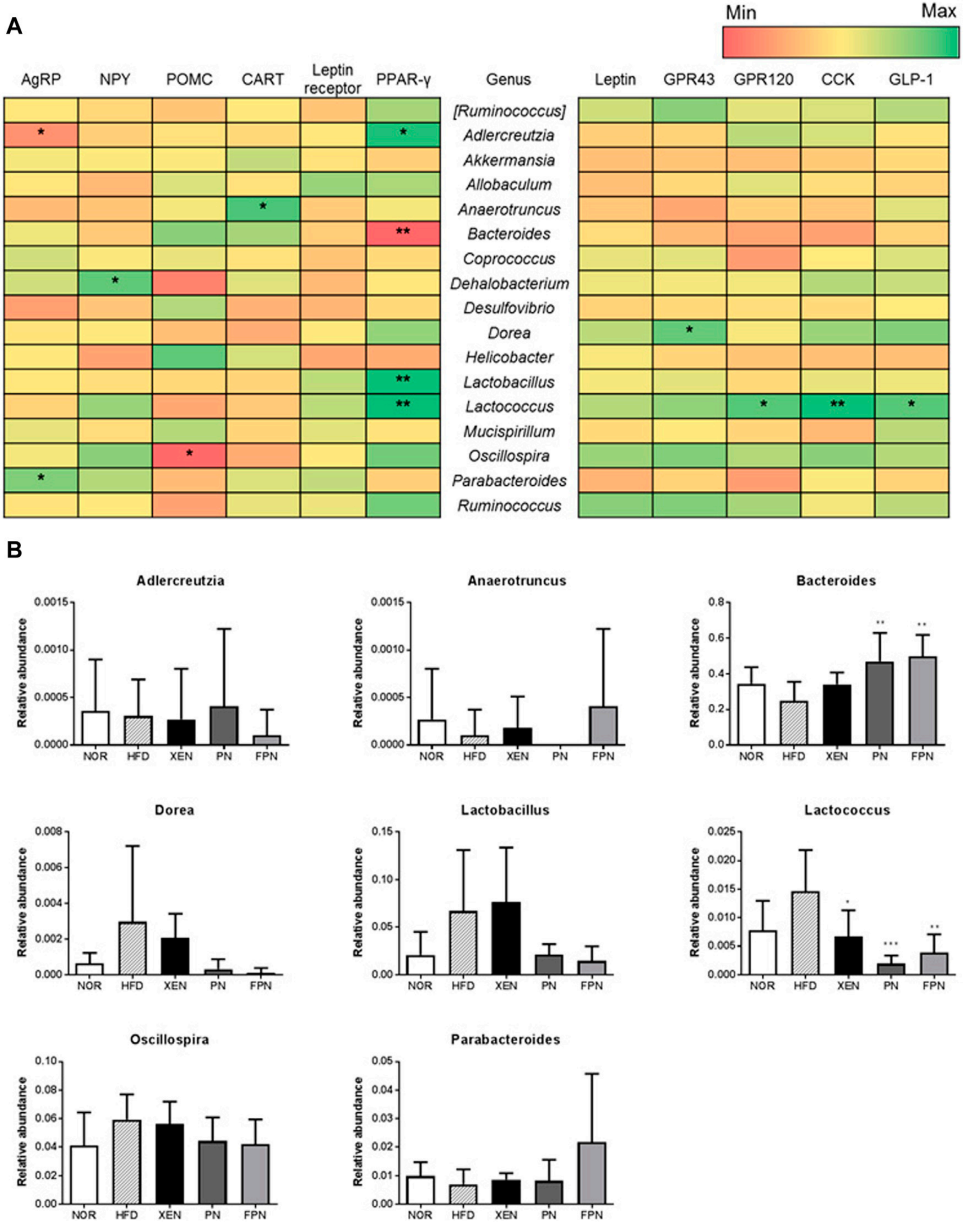
Correlation between the gut microbiota and hypothalamic and colonic gene expressions of the satiety hormone leptin, appetite regulators, and gut hormones in the HFD-induced mouse model after a treatment for 9 weeks. **(A)** Heatmap showing the correlation between the gut microbial population at the genus level and the expression levels of genes of appetite regulators and gut hormones. The Pearson correlation values were used for the matrix. *p < 0.05; **p < 0.01; ***p < 0.001. **(B)** The relative abundance of the bacterial genus correlated significantly with the gene expressions in the hypothalamus and colon. The data are expressed as the mean ± SEM (*n* = 7–8); *p < 0.05; **p < 0.01; ***p < 0.001; HFD group *vs.* treatment groups (XEN, PN and FPN).

The influence of appetite hormones on the gut microbiota was assessed by analyzing the correlation of the gene expression of the appetite markers with the gut microbiota communities. The results showed that at the genus level, *Adlercreutzia* was negatively correlated with the hypothalamic gene expression of AgRP, while *Parabacteroides* was positively correlated (Figure 9A). *Dehalobacterium* and *Oscillospira* were positively and negatively correlated with the hypothalamic mRNA levels of NPY and POMC, respectively. *Bacteroides* were negatively correlated with the gene expression of PPAR-γ in the hypothalamus. The relative abundance of this genus in the HFD group was increased significantly by PN and FPN treatment (Figure 9B). In contrast, *Adlercreutzia*, *Lactobacillus*, *Lactococcus*, and OTU 816470 were positively correlated with the hypothalamic mRNA level of PPAR-γ (Figure 9B; Supplementary Figures 4,5). Moreover, both *Lactococcus* and OTU 816470 were also positively correlated with the gene expression of GPR120, CCK, and GPL-1 in the colon and their relative abundances in the HFD group decreased significantly in response to the XEN, PN, and FPN treatment (Figure 9B; Supplementary Figure 5). *Dorea* was positively correlated with the colonic expression of the GPR43 gene.

## Discussion

The present study examined the anti-obesity impact of FPN and evaluated whether such activity could be mediated via the modulation of appetite and gut microbial population. PN contains saponin ginsenosides Rb1, Rg1, and Rd and notoginsenoside R1 as the major active compounds (Uzayisenga et al., 2014), which can be metabolized under the enzymatic action of β-glycosidases produced by the gut microbiota. Such bioconversion of ginsenosides to smaller deglycosylated forms, which are influenced mainly by the gut microbial composition of the host (Kim K.-A. et al., 2013), may modulate their biological activities and physiological actions (Park et al., 2017). Indeed, in the present study, PN showed a changed ginsenoside profile upon fermentation by the selected bacterial strain, *Lactobacillus plantarum* M41, which demonstrated markedly higher β-glucosidase activity. This concurs with previous reports showing the bioconversion of ginsenosides to their corresponding metabolites, such as Rg1, Rg3, compound K (CK), Rh1, and Rg2, as well as Rb1 and Rd by *L. plantarum*-mediated fermentation. The latter organic acid (acetic acid and lactic acid) has been proposed to function as an active metabolite that plays vital roles in muscle glycogen production, muscle production, and muscle fatigue, as well as in the modulation of energy production (Costa Leite et al., 2007) and energy homeostasis (Caesar et al., 2008).

In this study, both the food and calorie intake were decreased significantly in the HFD group upon treatment with FPN but not PN. The body weight, weights of the visceral fat and total fat, and the serum TG level were reduced significantly in the HFD-fed animals by both PN and FPN. The pharmacological effects of PN against hyperlipidemia are well documented (Zhao et al., 2017; Xu et al., 2018). The administration of PN to HFD-induced hyperlipidemic rats resulted in a significant decrease in the serum levels of total cholesterol, triglycerides, and low-density lipoprotein-cholesterol, an increase in the serum high-density lipoprotein-cholesterol levels, and a reduced hepatic level of HMG-CoA reductase (Xia et al., 2011). Furthermore, ginsenosides reduce the body weight, visceral fat, and body fat contents, as well as the serum TG level in the HFD-induced obese mice (Yao, 2020). In this study, however, the perigonadal fat weight and hepatic TG level were reduced significantly by FPN but not by PN. PPAR-γ plays a vital role in adipogenesis by maintaining the differentiated state of adipocytes (Rosen and Macdougald, 2006). LPL, a rate-limiting enzyme, regulates the hydrolysis of the TG core of circulating TG-rich lipoproteins, including chylomicrons, low-density lipoproteins, and very-low-density lipoproteins. In the present study, both PN and FPN downregulated the levels of hypothalamic PPAR-γ and adipose LPL gene expression significantly in the HFD group. PN saponins can regulate the lipid metabolism by inhibiting LPL and PPAR-γ (Duan et al., 2017). In the present study, the hepatic levels of PPAR-γ and LPL gene expression were reduced significantly by FPN but not PN.

To understand the molecular mechanisms underlying the inhibitory effects of FPN on food and calorie intake, this study examined the impact of PN and FPN on the gene expression of the major regulators of appetite and energy homeostasis, such as adipocyte hormone leptin and its receptor, neuropeptides, gut hormones, and the G-protein-coupled short-chain fatty acid receptors GPR43 and GPR120. The results showed that the mRNA levels of adipose leptin, hypothalamic neuropeptide NPY, and gut hormones (GLP1 and CCK) in the animals were increased significantly in response to HFD-feeding. Leptin is a key regulator of the mass of adipose tissue and body weight, and overexpression of the leptin gene has been observed in the subcutaneous adipose tissue of massively obese individuals (Sáinz et al., 2015). In contrast, NPY contributes to obesity by stimulating food intake and promoting weight gain (Spiegelman and Flier, 2001). GLP1 plays important roles in regulating postprandial glycemia through its insulinotropic action and gastric inhibitory effect (Holst, 2007). CCK, which is synthesized and released predominantly from the duodenum and jejunum, has been reported to reduce food intake dose-dependently (Perry and Wang, 2012). Fat is a potent CCK secretagogue, and high-fat diets increase the CCK levels (Swartz et al., 2010). These may lead to an impairment in lipid-induced CCK satiation signaling in the obese-prone condition and potentially contributing to hyperphagia and weight gain (Moehlecke et al., 2016). In the present study, the gene expression of CCK in the HFD group was downregulated significantly by both PN and FPN. In contrast, the mRNA levels of leptin and GLP1 in the HFD-fed animals were reduced significantly by a treatment with FPN but not PN. Overall, these results suggest that the inhibitory effects of FPN on the pathways related to appetite and energy intake as well as the anti-hyperlipidemic impact of this formulation are more potent than PN at an equivalent dose. In agreement with these findings, accumulating evidence also suggests the beneficial impact of fermentation on the anti-obesity properties of herbal medicines (Kim C. et al., 2013; Schneeberger et al., 2015; Hussain et al., 2016).

Generally, an overweight condition and obesity result from an imbalance in energy homeostasis (Ghanemi et al., 2018). On the other hand, accumulating evidence indicates that obesity is also associated with alterations in immunity, such as chronic low-grade inflammation, as evidenced by the increased levels of circulating pro-inflammatory cytokines (Lee et al., 2013) and inflammatory bowel disease (Lee et al., 2013; Pavelock et al., 2019). These findings concur with evidence indicating that HFD can induce the production of pro-inflammatory cytokines IL-1, IL-6, TNF-α, and MCP-1 in different tissues, which may be linked with obesity, insulin resistance, and other metabolic disorders. The colonic expression of TNF-α, MCP-1, and IL-1β and the colonic and hepatic mRNA levels of the inflammatory marker LBP in HFD-fed animals were downregulated significantly by PN and FPN. The protective effects of PN against a variety of chronic inflammatory conditions, including inflammatory bowel disease, arthritis, atherosclerosis, and Alzheimer's disease, have been well documented (Xu et al., 2018). In the present study, however, the expression of the LBP gene in adipose tissue was reduced significantly by FPN but not by PN. These results are in agreement with evidence indicating that the process of fermentation can enhance the anti-inflammatory activities of herbal medicines (Hussain et al., 2016), and the microbial transformation of ginsenosides improves their anti-inflammatory effects (Yu et al., 2017).

To understand the mechanism underlying the anti-inflammatory activities of PN and FPN better, the impact of these two formulations on the gene expression of anti-microbial peptides (AMPs), such as Pgc1α, Pla2g2α, and REg3γ, was also evaluated because AMPs protect against inflammation (Sun and Shang, 2015). The results showed that a HFD caused a significant increase in the expression of the Pgc1α gene but have no effect on the mRNA levels of the other three AMPs. Treatment of HFD-fed animals with both PN and FPN did not modulate the gene expression of Pgc1α.

The gut microbiota is an important factor for obesity because it plays significant roles in maintaining the nutrients and energy balance, lipid metabolism, and immune response in the host (Turnbaugh et al., 2006). The impact of the gut microbiota differs according to itscomposition and population (Wang et al., 2018). Therefore, diversity is a critical factor in predicting the potential roles of the gut microbiota. In the unweighted PCoA of the beta-diversity assessment, distinguishable clustering of the gut microbial population was demonstrated by all the experimental groups with clear segregation of the entire treated groups from the normal one. This analysis further showed that the principal component F1 and F2 scores in the HFD group were significantly higher and lower, respectively, than the normal group. In addition, both Chao1 and the observed OTUs, which are indices of α-diversity that measure the species richness, were significantly lower in the HFD group than the normal group. Overall, these findings show that the changes in the gut microbial diversity are a response to HFD feeding. Diet is one of the critical factors influencing the gut microbial composition (Kang et al., 2017). Accumulating evidence suggests that a HFD alters the gut microbial diversity in the animals. In the present study, both F1 and F2 scores of unweighted PCoA in HFD-fed animals were changed significantly in response to treatment with either PN or FPN. In particular, these two scores were improved significantly in the HFD-fed animals upon treatment with FPN. In contrast, α-diversity analysis showed that PN and FPN had no significant effect on Chao1 in the HFD group. On the other hand, treatment of the HFD-fed animals with PN improved the observed OTUs significantly but not with FPN. Overall, these data indicate the differential responses of the diversity parameters in the HFD group to the herbal formulations used in the present study.

At the phylum level, the highest relative abundance of Firmicutes (F) and the lowest Bacteroidetes (B) population were observed in the animals in response to HFD feeding, reflecting the significantly higher F/B ratio in the HFD group compared to the normal group. Accumulating evidence suggests that an increased F/B ratio is associated with obesity (Koliada et al., 2017). Indeed, the genus *Bacteroides* was negatively correlated with the gene expression of hypothalamic PPAR-γ, which contributes to HFD-induced leptin resistance (Ryan et al., 2011), a condition that leads to the development of obesity and metabolic disorders, such as insulin resistance and dyslipidemia (Gruzdeva et al., 2019). In the present study, the HDF + FPN-treated mice showed the highest relative abundance of Bacteroidetes among the experimental groups. They demonstrated a significantly higher population of *Bacteroides* compared to the HFD-fed animals. Furthermore, treatment of the HFD-fed mice with PN and FPN decreased the population of genus *Lactococcus* and OTU 816470 significantly, which were positively correlated with hypothalamic PPAR-γ and gut hormones CCK and GLP1.

In conclusion, these results suggest that both PN and FPN have a beneficial impact on HFD-induced obesity. Compared to PN, however, FPN exerted greater anti-hyperlipidemic and anti-inflammatory effects and is more potent in suppressing the signaling pathways related to appetite and energy intake. The changed ginsenoside and organic acid profile, and altered gut microbial composition appear to be the major factors contributing to the properties of FPN.

## Data Availability

The raw data of the sequence is available in the European Nucleotide Archive with the accession numbers ERP119284.

## Abbreviations

ANOVA: one-way analysis of variance; AMP: anti-microbial peptides; CK: compound K; ESI: electrospray ionization; FPN: fermented *Panax notoginseng*; GAPDH: glyceraldehyde-3-phosphatase dehydrogenase; GLP-1: glucagon-like peptide-1; HFD: high-fat diet; HPLC: high-performance liquid chromatography; LAB: lactic acid bacteria; LEfSe: LDA effect size; NOR: normal group; OTU: operational taxonomic units; PN: *Panax notoginseng*; p-NPG: β-D-glucopyranoside; QIIME: quantitative insights into microbial ecology; RT-qPCR: real-time quantitative polymerase chain reaction; TOF/MS: time of flight/mass spectrometry; UPLC: ultra performance liquid chromatography.
