# Supplementary material for: Anti-Obesity Effect of Fermented Panax notoginseng Is Mediated Via Modulation of Appetite and Gut Microbial Population
Source: Front Pharmacol. 2021 Jul 26;12:665881. doi: 10.3389/fphar.2021.665881 (PMC8350340; doi:10.3389/fphar.2021.665881)
Supplement: Supplementary file 1 [file DataSheet1.PDF]

Supporting data

Materials and methods

Hematoxylin and eosin (H&E) staining

The colonic tissue slices were fixed in 4% paraformaldehyde overnight and embedded in paraffin blocks for histological processing. The tissues were sectioned to 5 μm thickness using a microtome (Leica, Nussloch, Germany) and placed on positively charged glass slides (Leica Biosystem, Richmond, IL, USA), followed by deparaffinization with xylene (Duksan, Ansan, Gyunggido, South Korea) and dehydration through an increasing ethanol concentration gradient. The tissue sections were stained with hematoxylin solution (EMD Millipore, Darmstadt, Germany) and eosinY solution (0.5%) alcoholic (Biosciences, St. Louis, MO, USA) and then mounted on cover slips. The stained tissues were examined under an inverted light microscope (Olympus, Tokyo, Japan) at 100×, 200×, and 400× magnifications. Microscopic images were captured using a digital camera (Olympus, Tokyo, Japan).

Tables

Table S1. Nucleotide sequences of primers used in quantitative real-time PCR

Gene	Forward	Reverse
PPAR γ	GCC TGT CTG TCG GGA TGT	GGC TTC GTG GAT TCT CTT
LPL	TTG CCC TAA GGA CCC CTG AA	ACA GAG TCT GCT AAT CCA GGA AT
Leptin	GCC GGT GTG AGT TTT CAG TCA	CCT AAG GGT GGA TCG GGT TT
Leptin receptor	TGA GGT ATC ACA GGC GCA GCC T	ACG CAG TTT TTG GGC CTC AGA CGT
POMC	TGC TTC AGA CCT CCA TAG ATG TGT	GGA TGC AAG CCA GCA GGT T
CART	CTG CAA TTC TTT CCT CTT GAA GTG	GGG AAT ATG GGA ACC GAA GGT
AgRP	CGG AGG TGC TAG ATC CAC AGA	AGG ACT CGT GCA GCC TTA CAC
NPY	TAC TCC GCT CTG CGA CAC TAC A	AAT CAG TGT CTC AGG GCT GGA T
GLP-1	GGC ACA TTC ACC AGC GAC TAC	CAA TGG CGA CTT CTT CTG GG
CCK	GCA CTG CTA GCG CGA TAC ATC	CCA GGC TCT GCA GGT TCT TAA G
GPR43	ACA GTG GAG GGG ACC AAG AT	GGG GAC TCT CTA CTC GGT GA
GPR120	GTG CCG GGA CTG GTC ATT GTG	TTG TTG GGA CAC TCG GAT CTG G
TNF α	GGT GAA GGG AAT GGG TAT	GGT CAC TGT CCC AGC ATC TT
MCP1	AAG AGA TCA GGG AGT TTG CT	CTG CCT CCA TCA ACC ACT TT
IL-1β	GTG GCA ATG AGG ATG ACT TGT TC	TTG CTG TAG TGG TCG GAG
Pgc1a	AGG ATT CCC CCA AGG ATG CCA C	CAG CCG TTT CTG ACA GGA GTT CTG G
Pla2g2a	AGG ATT CCC CCA AGG ATG CCA C	CAG CCG TTT CTG ACA GGA GTT CTG G
Reg3g	TTC CTG TCC TCC ATG ATC AAA	CAT CCA CCT CTG TTG GGT TC
LBP	GTC CTG GGA ATC TGT CCT TG	CCG GTA ACC TTG CTG TTG TT
GAPDH	GAC ATC AAG AAG GTG GTG AAG CAG	ATA CCA GGA AAT GAG CTT GAC AAA

Table S2. Concentration of acetic and lactic acids in PN and FPN

Organic acid (mM)	PN	FPN
lactic acid	0.0	246.3
acetic acid	0.0	28.0

Table S3. Permutational multivariate analysis of variance (PERMANOVA) of β-diversity measured by UniFrac to show the inter-group differences in gut microbial composition.

UniFrac	pairs	F value	R2	p.value
Unweighted	NOR vs XEN	0.856497	0.057651	0.789
	NOR vs PN	1.238953	0.087011	0.102
	NOR vs HFD	2.425156	0.157221	**0.001**
	NOR vs FPN	2.106609	0.139449	**0.001**
	XEN vs PN	1.162996	0.082115	0.14
	XEN vs HFD	2.470183	0.159674	**0.002**
	XEN vs FPN	1.863321	0.125364	**0.002**
	PN vs HFD	2.658551	0.181365	**0.001**
	PN vs FPN	2.014031	0.143715	**0.001**
	HFD vs FPN	0.914644	0.070822	0.637
Weighted	NOR vs XEN	0.509582	0.03512	0.763
	NOR vs PN	5.849702	0.310334	**0.002**
	NOR vs HFD	1.051793	0.074851	0.357
	NOR vs FPN	1.096866	0.077809	0.334
	XEN vs PN	2.68953	0.171422	0.085
	XEN vs HFD	0.852681	0.061553	0.386
	XEN vs FPN	0.357822	0.026787	0.741
	PN vs HFD	3.552781	0.228434	**0.04**
	PN vs FPN	1.373662	0.102714	0.238
	HFD vs FPN	0.527773	0.042128	0.564

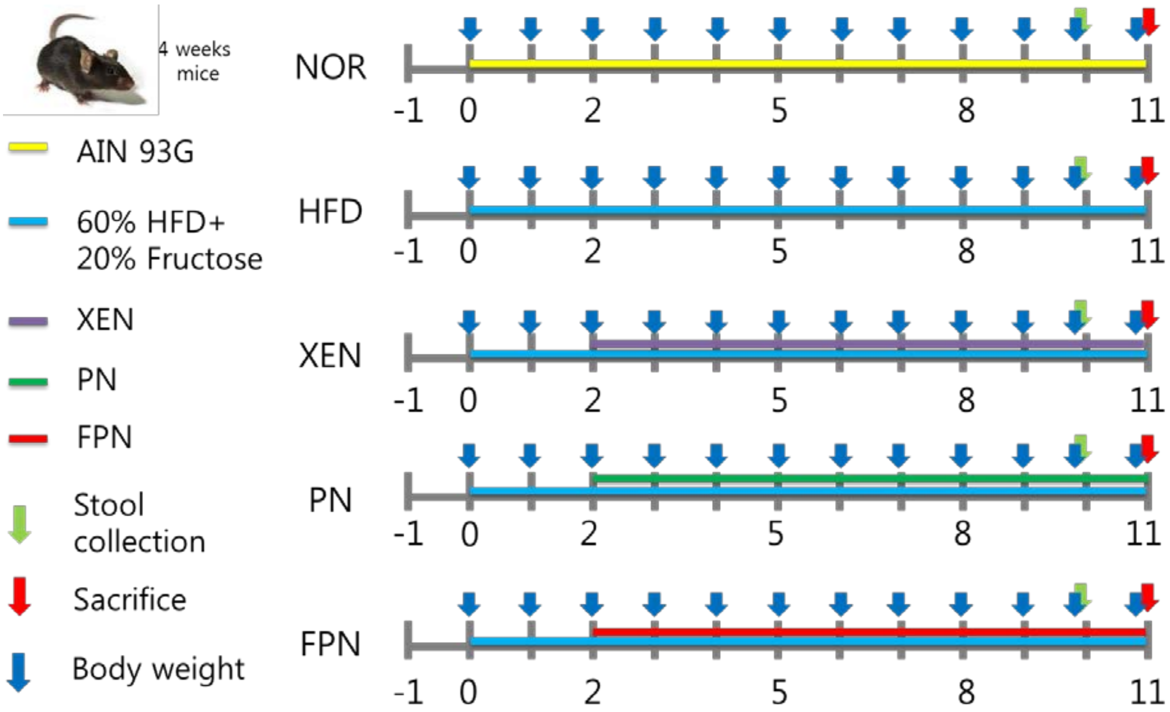

Figure S1

Animal experimental schedule

A

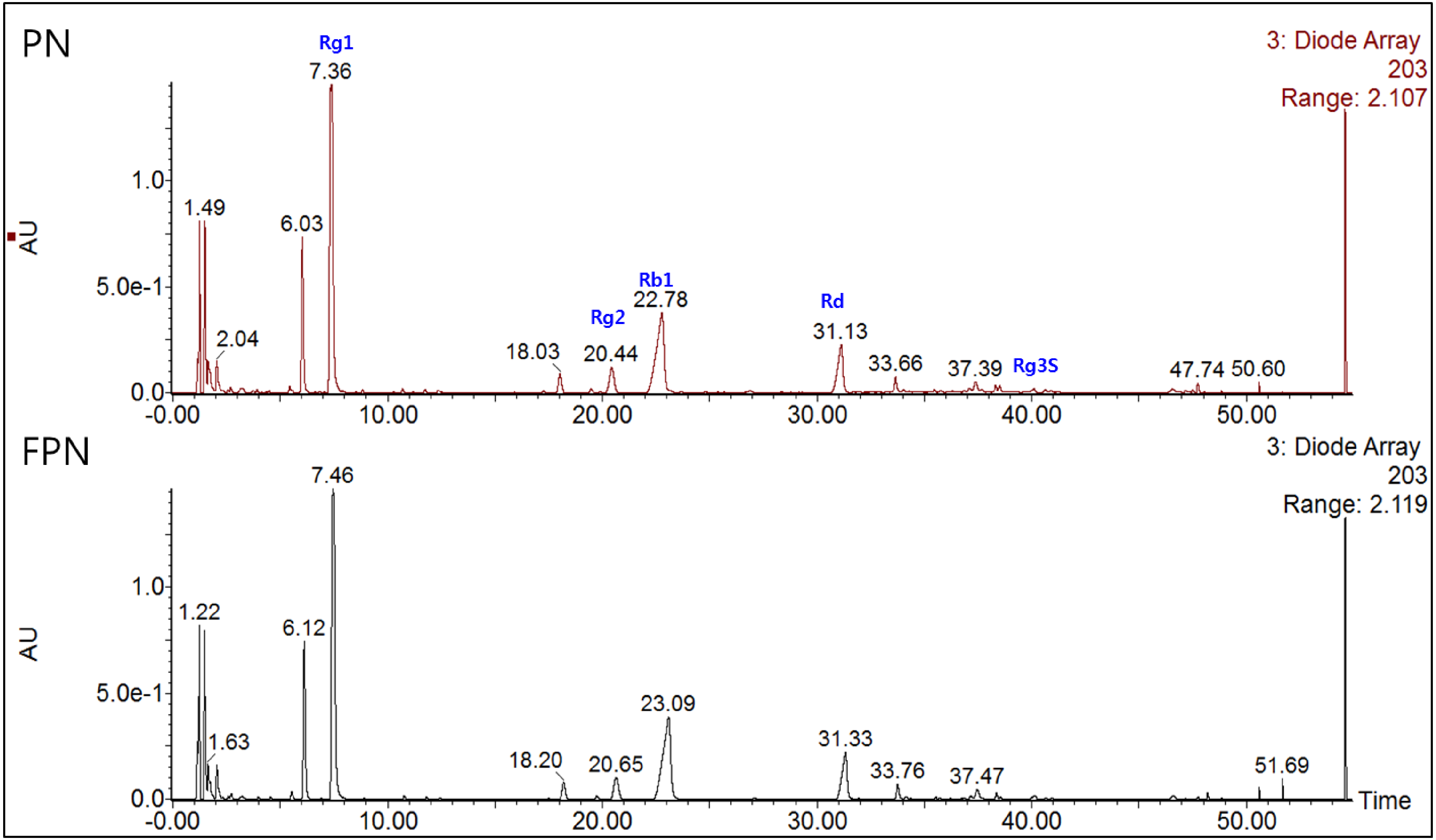

PN
3: Diode Array
203
Range: 2.107
AU
1.0
5.0e-1
0.0
Rg1
7.36
1.49
6.03
2.04
18.03
Rg2
20.44
Rb1
22.78
Rd
31.13
33.66
37.39
Rg3S
47.74
50.60
-0.00
10.00
20.00
30.00
40.00
50.00
FPN
3: Diode Array
203
Range: 2.119
AU
1.0
5.0e-1
0.0
7.46
1.22
6.12
1.63
18.20
20.65
23.09
31.33
33.76
37.47
51.69
Time
-0.00
10.00
20.00
30.00
40.00
50.00

B

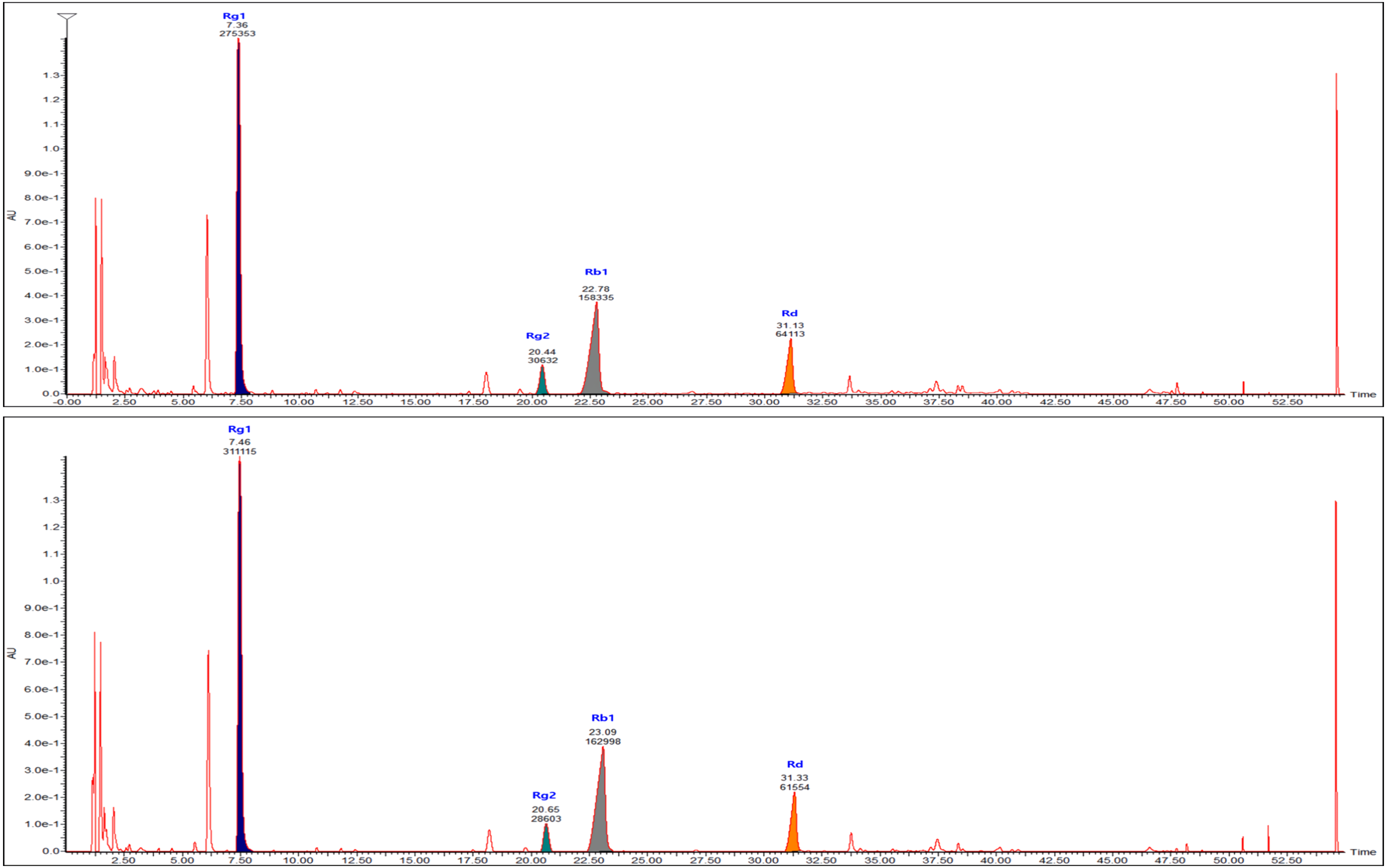

Rg1
7.36
275353
Rg2
20.44
30632
Rb1
22.78
158335
Rd
31.13
64113
AU
Time
Rg1
7.46
311115
Rg2
20.65
28603
Rb1
23.09
162998
Rd
31.33
61554
AU
Time

C

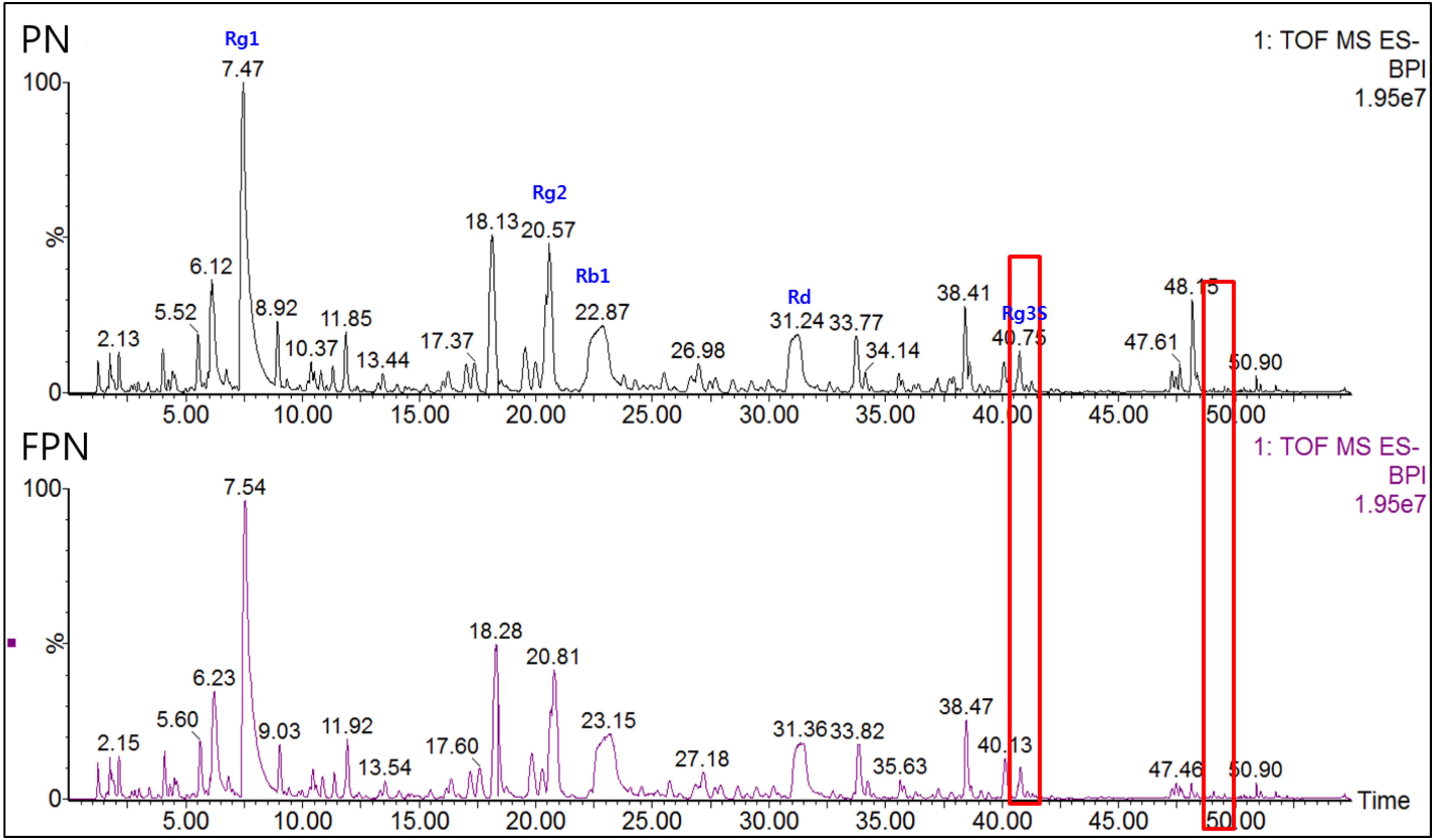
PN
1: TOF MS ES-
BPI
1.95e7
Rg1
7.47
Rg2
18.13
20.57
Rb1
22.87
Rd
31.24
33.77
34.14
38.41
Rg3S
40.75
48.15
47.61
50.90
6.12
5.52
2.13
8.92
10.37
11.85
13.44
17.37
26.98
100
%
0
5.00
10.00
15.00
20.00
25.00
30.00
35.00
40.00
45.00
50.00
FPN
1: TOF MS ES-
BPI
1.95e7
7.54
6.23
5.60
2.15
9.03
11.92
13.54
17.60
18.28
20.81
23.15
27.18
31.36
33.82
35.63
38.47
40.13
47.46
50.90
Time

D

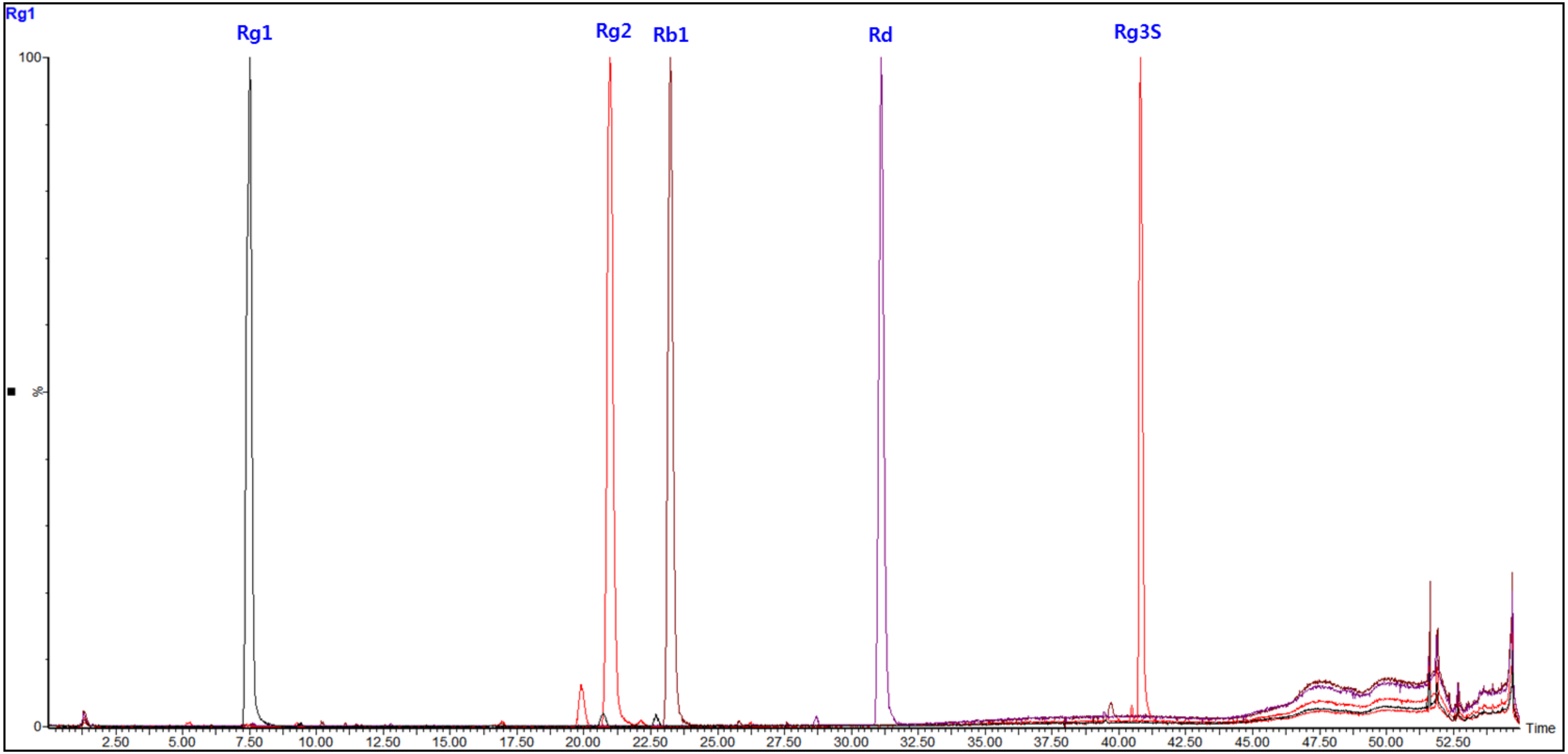
Rg1
Rg1
Rg2
Rb1
Rd
Rg3S
100
%
0
2.50
5.00
7.50
10.00
12.50
15.00
17.50
20.00
22.50
25.00
27.50
30.00
32.50
35.00
37.50
40.00
42.50
45.00
47.50
50.00
52.50
Time

E

48.15 min MASS chromatography and MASS spectrum

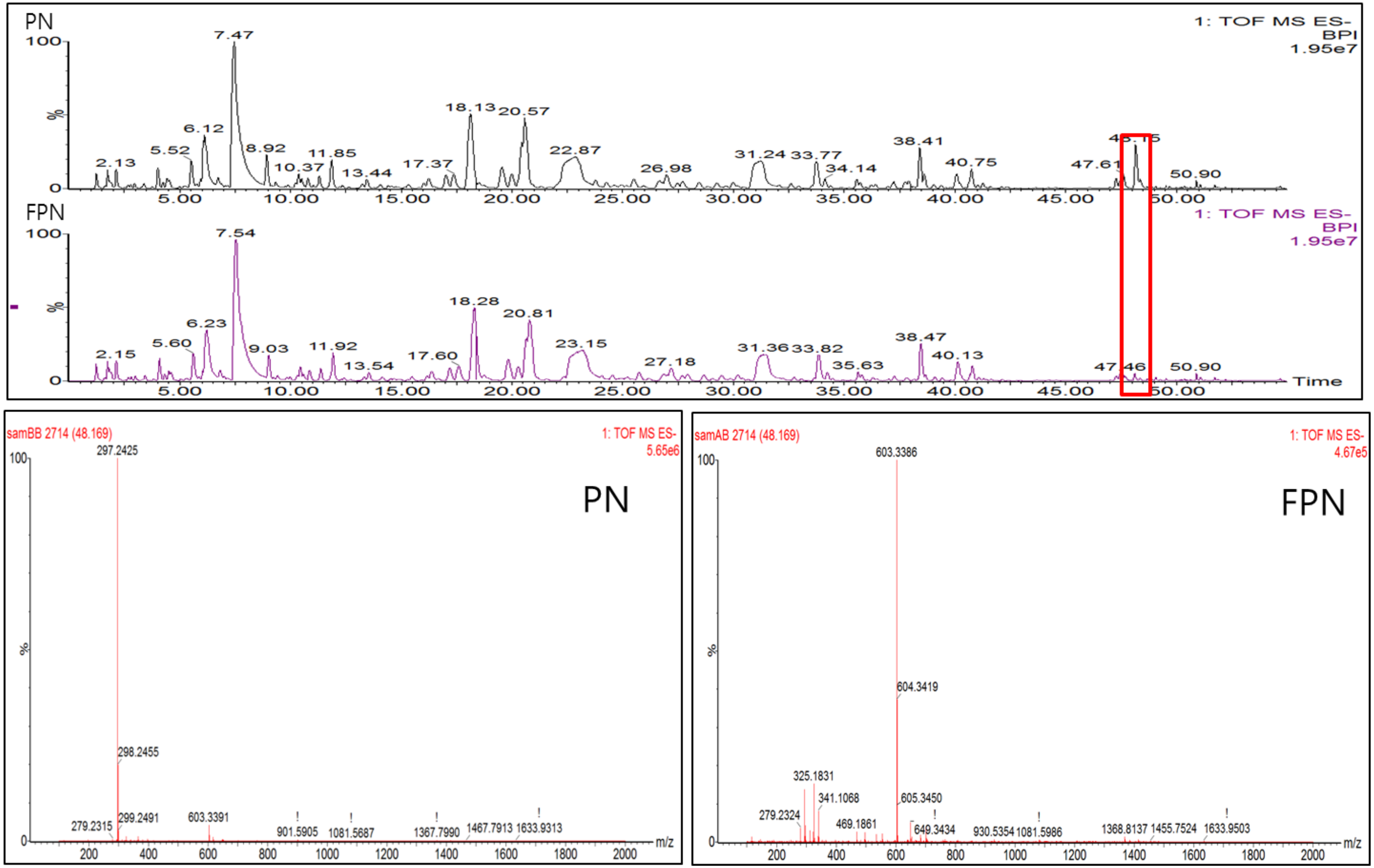

F

Rg1 MASS chromatography and MASS spectrum

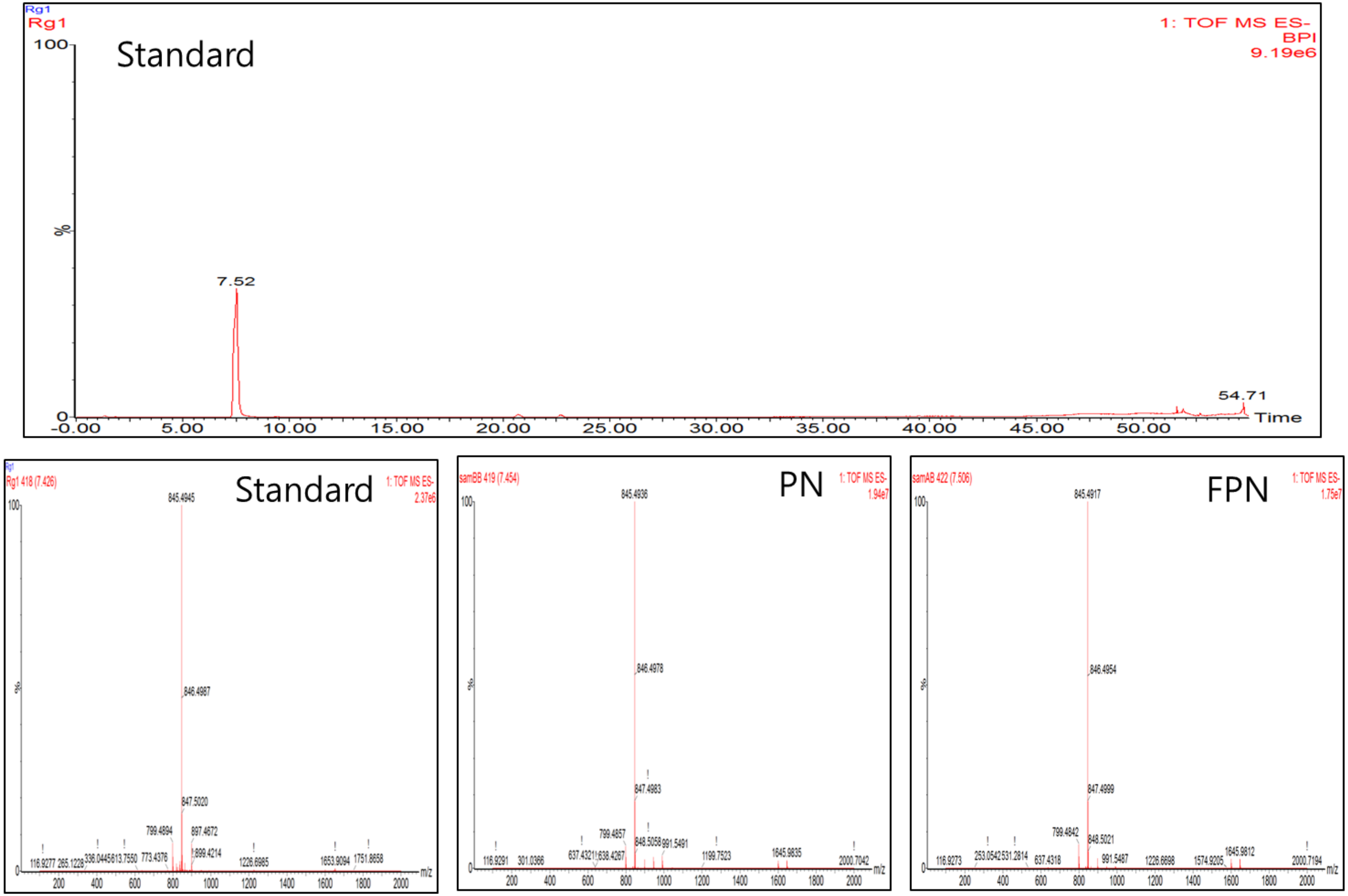

G

Rg2S MASS chromatography and MASS spectrum

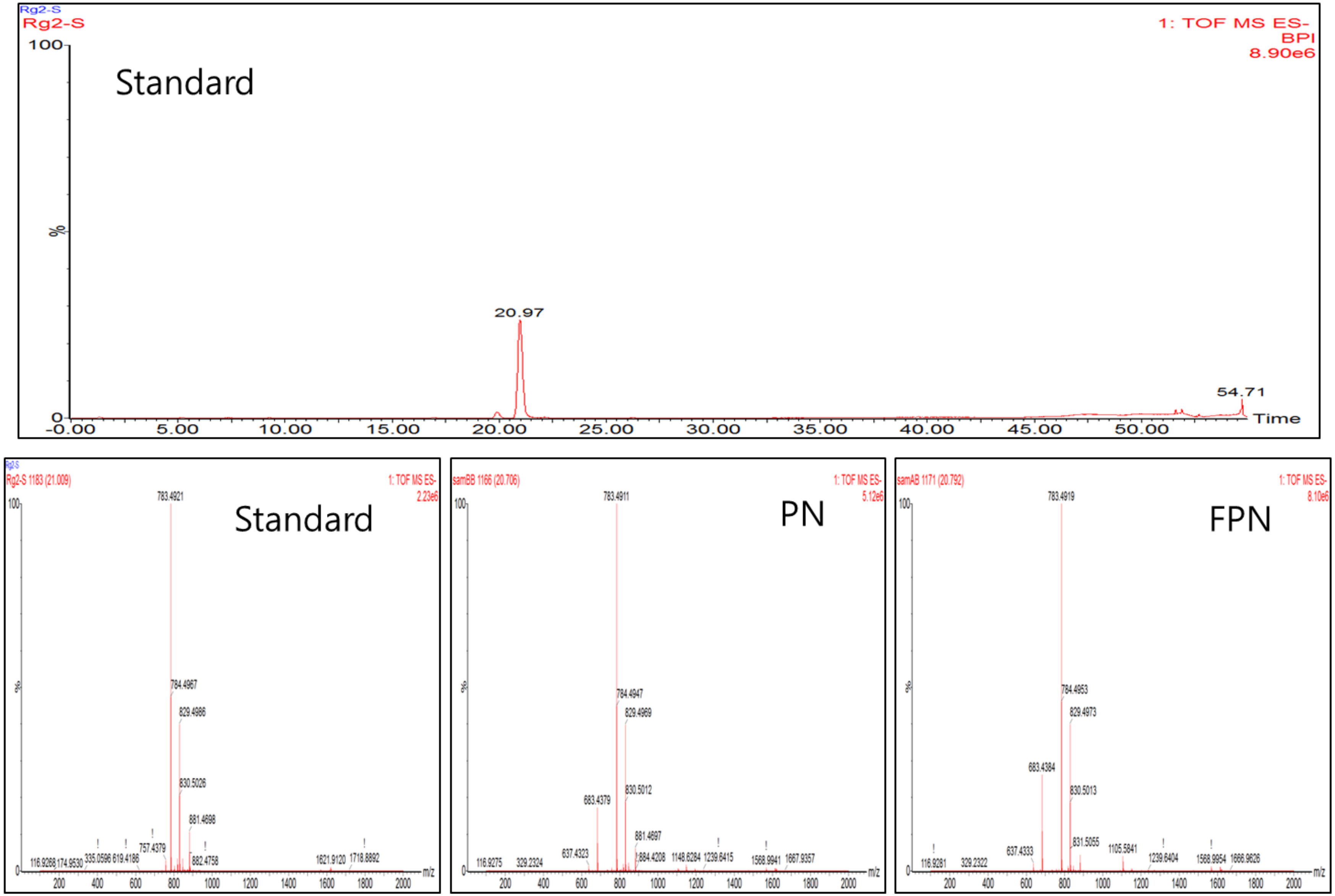

H

Rb1 MASS chromatography and MASS spectrum

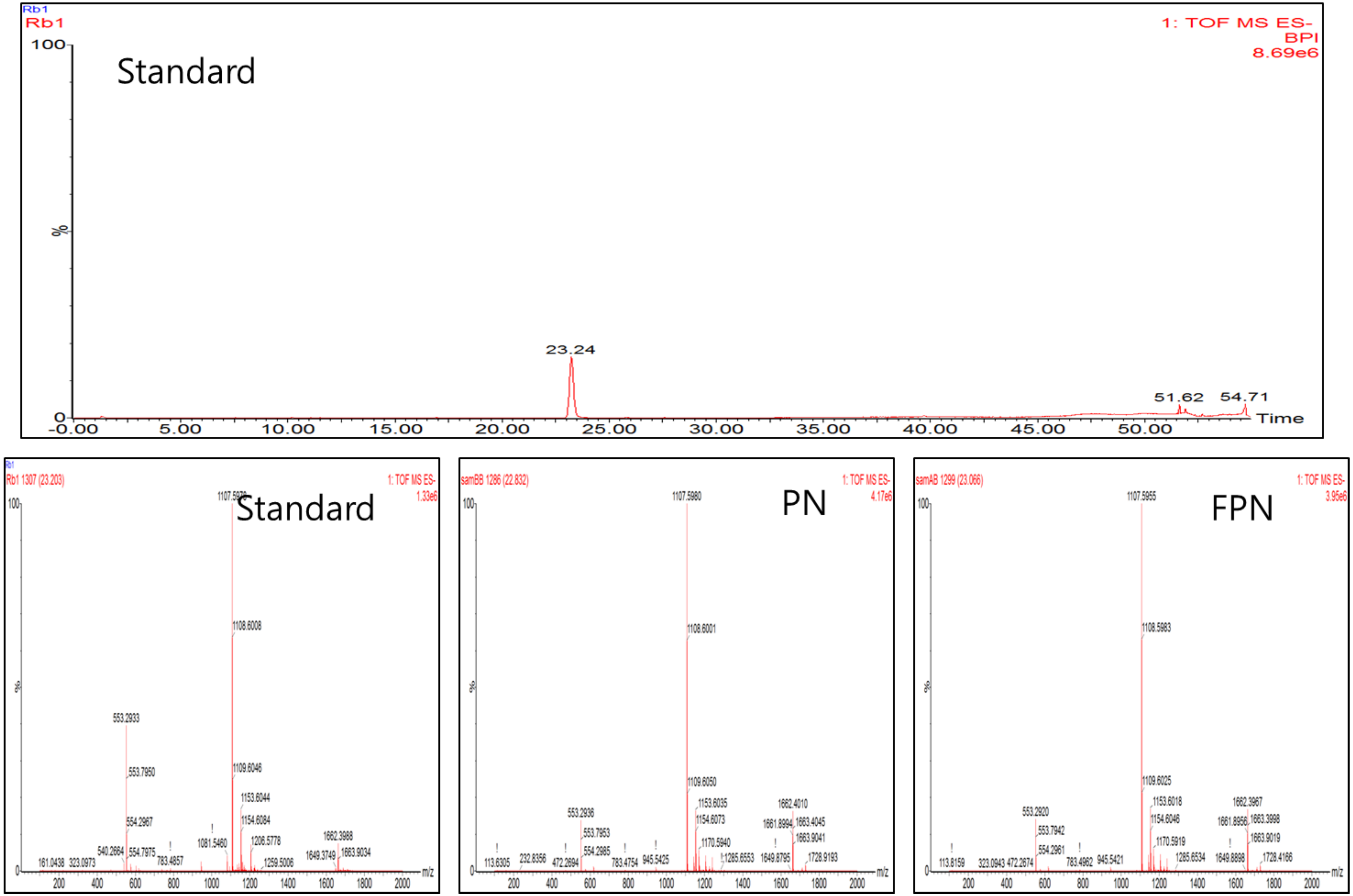

I

Rd MASS chromatography and MASS spectrum

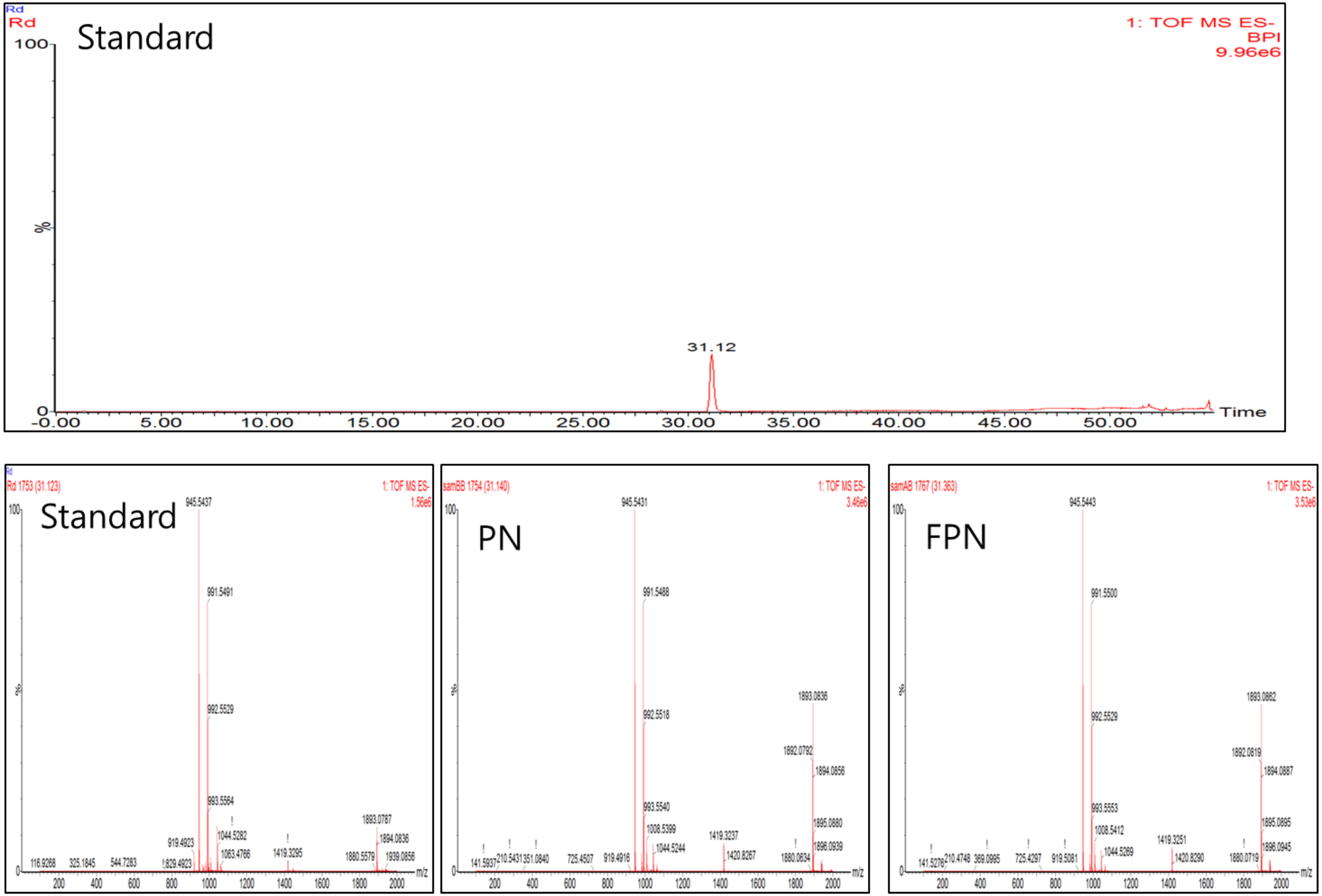

J

Rg3S MASS chromatography and MASS spectrum

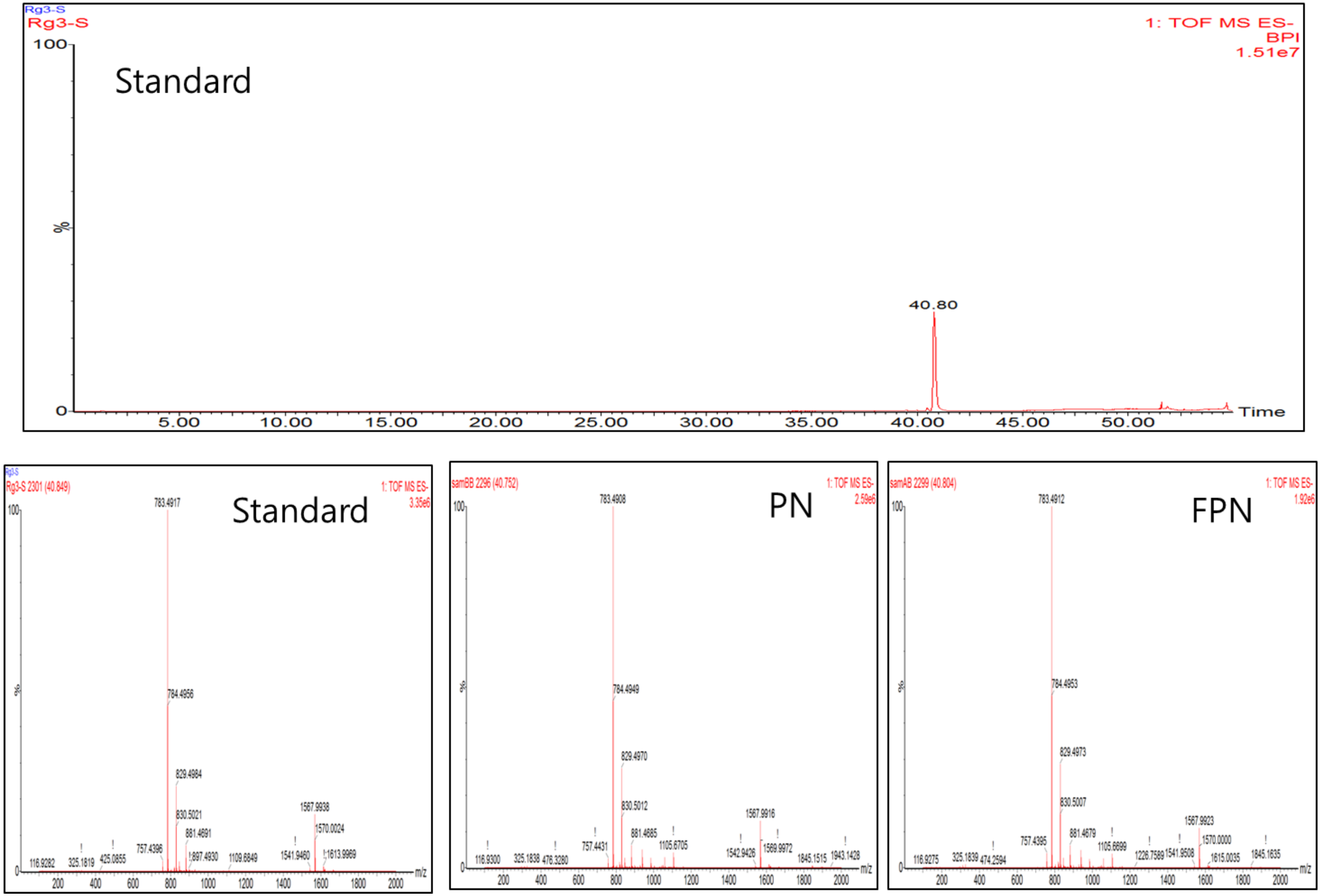

Figure S2. UPLC-TOF-MS analysis of ginsenosides Rg1, Rg2, Rb1, Rd and Rg3S of PN and FPN and corresponding standards.

(A) UPLC UV chromatogram of PN and FPN (B) UPLC UV chromatogram of ginsenoside peaks of PN and FPN depicting peak areas, (C) Mass spectra of PN and FPN, (D) Mass spectra of ginsenoside standards and (E) Mass spectra of 48.169 min peak in PN and FPN as well as (F) Rg1, (G) Rg2, (H) Rb1, (I) Rd and (J) Rg3S in standards, PN, and FPN.

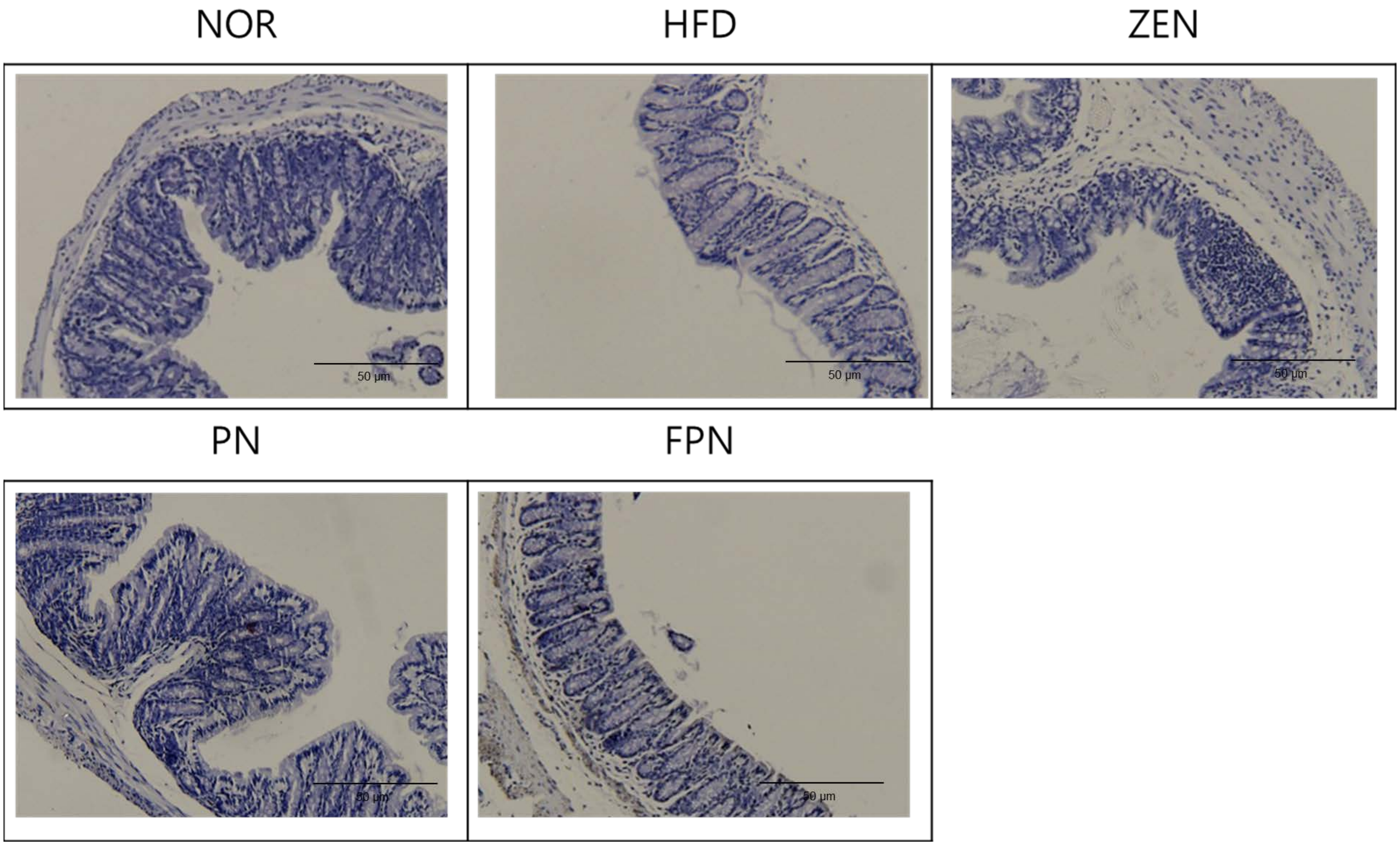

Figure S3

Histological evaluation of the H&E-stained colonic tissue of an HFD-induced mouse model either untreated or treated with different agents. The tissue sections were visualized under light microscope with 400X magnification (scale bar 50 μm).

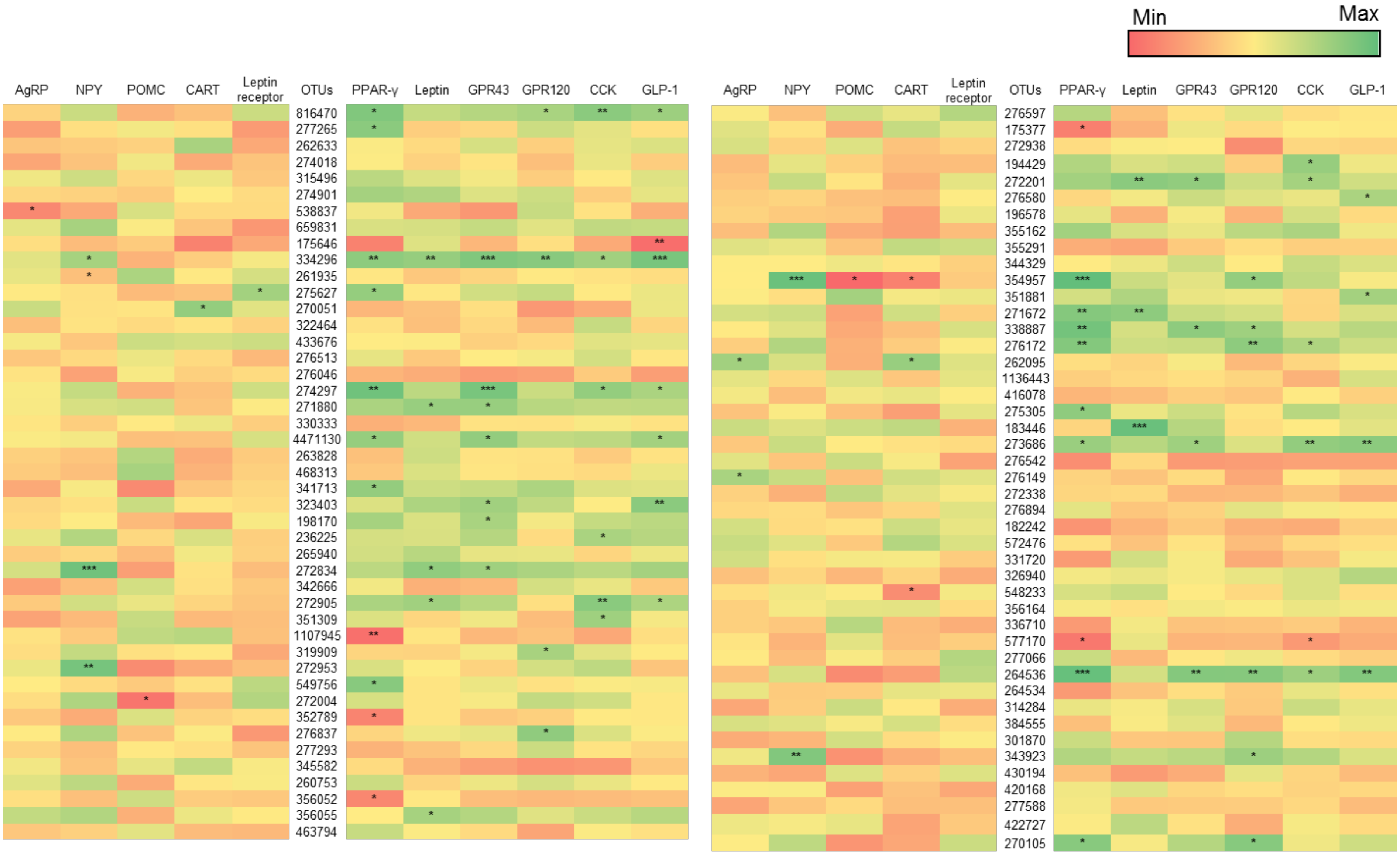
Min
Max
AgRP
NPY
POMC
CART
Leptin receptor
OTUs
PPAR-γ
Leptin
GPR43
GPR120
CCK
GLP-1
816470
277265
262633
274018
315496
274901
538837
659831
175646
334296
261935
275627
270051
322464
433676
276513
276046
274297
271880
330333
4471130
263828
468313
341713
323403
198170
236225
265940
272834
342666
272905
351309
1107945
319909
272953
549756
272004
352789
276837
277293
345582
260753
356052
356055
463794
AgRP
NPY
POMC
CART
Leptin receptor
OTUs
PPAR-γ
Leptin
GPR43
GPR120
CCK
GLP-1
276597
175377
272938
194429
272201
276580
196578
355162
355291
344329
354957
351881
271672
338887
276172
262095
1136443
416078
275305
183446
273686
276542
276149
272338
276894
182242
572476
331720
326940
548233
356164
336710
577170
277066
264536
264534
314284
384555
301870
343923
430194
420168
277588
422727
270105

Figure S4

Correlation between the OUT of gut microbiota and gene expression of ppar-γ, appetite, and gut hormone on the hypothalamus and colon in the HFD-induced mouse model after treatment for nine weeks.

Heatmap of the correlation between the alterations in gut microbial population at OTU level and the gene expression changes related to the appetite and gut hormones. The Pearson correlation values were used for the matrix. *$P < 0.05$;**$P < 0.01$; ***$P<0.001$.

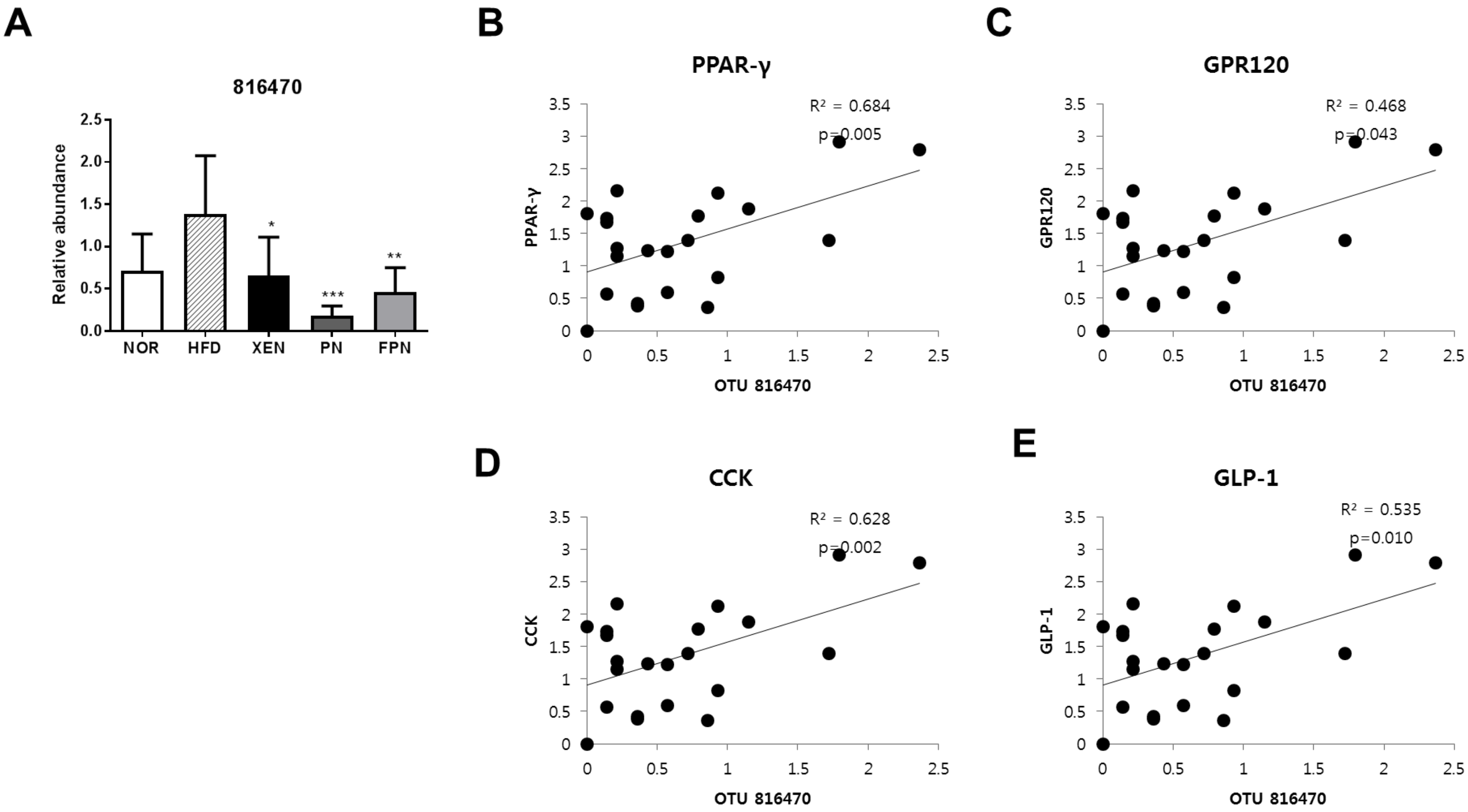

Figure S5

Relative abundance of OUT 816470 and correlation analysis between OUT 816470 and gene expression of PPAR-γ on the hypothalamus and gut hormone on the colon in an HFD induced mouse model after a treatment for nine weeks.

(A) Relative abundance of OUT 816470 of gut microbiota; correlation analysis between OUT 816470 and (B) PPAR-γ on the hypothalamus, (C) GPR120, (D) CCK, and (E) GLP-1 on the colon.
